# Hesperidin Bioavailability Is Increased by the Presence of 2S-Diastereoisomer and Micronization—A Randomized, Crossover and Double-Blind Clinical Trial

**DOI:** 10.3390/nu14122481

**Published:** 2022-06-15

**Authors:** Anna Crescenti, Antoni Caimari, Juan María Alcaide-Hidalgo, Roger Mariné-Casadó, Rosa M. Valls, Judit Companys, Patricia Salamanca, Lorena Calderón-Pérez, Laura Pla-Pagà, Anna Pedret, Antoni Delpino-Rius, Pol Herrero, Iris Samarra, Lluís Arola, Rosa Solà, Josep M. Del Bas

**Affiliations:** 1 Eurecat, Centre Tecnològic de Catalunya, Unitat de Nutrició i Salut, Av/de la Universitat, 1, 43204 Reus, Spain; juanmaria.alcaide@eurecat.org (J.M.A.-H.); roger.marine@eurecat.org (R.M.-C.); judit.companys@eurecat.org (J.C.); lorena.calderon@eurecat.org (L.C.-P.); laurapla.dn@gmail.com (L.P.-P.); lluis.arola@urv.cat (L.A.); josep.delbas@eurecat.org (J.M.D.B.); 2 Eurecat, Centre Tecnològic de Catalunya, Biotechnology Area, Av/de la Universitat, 1, 43204 Reus, Spain; antoni.caimari@eurecat.org; 3 Oxidation and Cardiovascular Diseases Group (NFOC-Salut), Facultat de Medicina i Ciències de la Salut, Functional Nutrition, Universitat Rovira i Virgili, Sant Llorenç, 21, 43201 Reus, Spain; patricia.salamanca@urv.cat (P.S.); anna.pedret@urv.cat (A.P.); rosa.sola@urv.cat (R.S.); 4 Eurecat, Centre Tecnològic de Catalunya, Unitat de Ciències Òmiques, Av/de la Universitat 1, 43204 Reus, Spain; antoni.delpino@eurecat.org (A.D.-R.); pol.herrero@agilent.com (P.H.); iris.samarra@eurecat.org (I.S.); 5 Nutrigenomics Research Group, Department of Biochemistry and Biotechnology, Campus Sescelades, Universitat Rovira i Virgili, 43007 Tarragona, Spain; 6 Internal Medicine Service, Hospital Universitari Sant Joan de Reus, Av/del Doctor Josep Laporte, 2, 43204 Reus, Spain

**Keywords:** hesperidin, hesperetin, bioavailability, metabolites, catabolites, urinary excretion, hesperidin diastereoisomers, micronization

## Abstract

Hesperidin is a flavanone abundantly found in citrus fruits for which health beneficial effects have been reported. However, hesperidin shows a low bioavailability among individuals. The aim of this study was to evaluate the effects of the micronization process and 2R- and 2S-hesperidin diastereoisomers ratio on hesperidin bioavailability. In a first phase, thirty healthy individuals consumed 500 mL of orange juice with 345 mg of hesperidin, and the levels of hesperidin metabolites excreted in urine were determined. In the second phase, fifteen individuals with intermediate hesperidin metabolite levels excreted in urine were randomized in a crossover, postprandial and double-blind intervention study. Participants consumed 500 mg of the hesperidin-supplemented Hesperidin epimeric mixture (HEM), the micronized Hesperidin epimeric mixture (MHEM) and micronized 2S-Hesperidin (M2SH) in each study visit with 1 week of washout. Hesperidin metabolites and catabolites were determined in blood and urine obtained at different timepoints over a 24 h period. The bioavailability—relative urinary hesperidin excretion (% of hesperidin ingested)—of M2SH (70 ± 14%) formed mainly by 2S-diastereoisomer was significantly higher than the bioavailability of the MHEM (55 ± 15%) and HEM (43 ± 8.0%), which consisted of a mixture of both hesperidin diastereoisomers. Relative urinary excretion of hesperidin metabolites for MHEM (9.2 ± 1.6%) was significantly higher compared to the HEM (5.2 ± 0.81%) and M2SH (3.6 ± 1.0%). In conclusion, the bioavailability of 2S-hesperidin extract was higher compared to the standard mixture of 2S-/2R-hesperidin extract due to a greater formation of hesperidin catabolites. Furthermore, the micronization process increased hesperidin bioavailability.

## 1. Introduction

The flavonoid hesperidin (hesperetin-7-O-rutinoside) is the β-glycosylated form of the flavanone hesperetin (aglycone form). This polyphenol is abundantly found in citrus fruits and is the major flavonoid present in sweet oranges (*Citrus sinensis*) and orange juice [1]. Hesperidin has been reported to provide health beneficial effects—including anticarcinogenic [2], antioxidant [3], anti-inflammatory [4,5], hypocholesterolemic [3,5] and hypoglycemic [6] properties—as well as beneficial effects for several diseases such as cancer and neurodegenerative and cardiovascular diseases [2,3,4,6,7,8,9].

Hesperidin has a chiral carbon in position 2 that generates two diastereoisomers, 2R- and 2S-. The predominant form in nature of hesperidin is the 2S-diastereoisomer, with a ratio of 8/92 for 2R-/2S-hesperidin diastereoisomers in fresh fruit products [10]. However, most current hesperidin products are commercially available as a mixture of both diastereoisomers due to the process of extracting hesperidin from natural fruit sources [11,12]. Several studies have reported that the two hesperidin diastereoisomers may present differences in transport, metabolism, biological effects [10,11] and kinetics in plasma and urine [12,13].

The hesperidin molecule is conjugated to the rutinose disaccharide formed by the sugar molecules rhamnose and glucose. The presence of a rutinose moiety is responsible for most of the ingested hesperidin having to be metabolized and absorbed in the colon since it cannot be hydrolyzed by the β-glucosidases of the small intestine [14,15]. In the colon, hesperidin is metabolized and hydrolyzed by the intestinal microbiota—mainly to the aglycone form hesperetin—before being absorbed by the colonocytes [15]. Once absorbed, hesperetin in the colon is metabolized by phase II enzymes—UDP-glucuronosyl transferases and sulfotransferases—to be conjugated with glucuronic acid or sulphate and other subsequent conjugations, and to finally be released into the bloodstream [16,17]. In addition, a substantial proportion of hesperetin is further metabolized by the microbiota present in the colon, generating bioavailable and highly specific hesperetin catabolites such as 3-(3′-hydroxy-4′-methoxyphenyl) propanoic acid (HMPPA) and also less specific catabolites such as hippuric acid, 4-hydroxyhippuric acid and 3-Hydroxy-3-(3′-hydroxyphenyl) propanoic acid, which are also formed from other phenolic compounds [16,17,18].

The results of several interventional clinical studies evaluating the bioavailability of hesperidin show a low bioavailability and highly variable among individuals [18,19,20]. In fact, it has been proposed individuals may be stratified as high-, intermediate- or low-hesperidin metabolite excretors [21,22,23,24,25]. However, several studies show that the bioavailability of hesperidin increases considerably if catabolites generated by the intestinal microbiota are considered [18,22,26,27]. Furthermore, hesperidin presents a low solubility that may affect its absorption and therefore its bioavailability [28]. In this sense, hesperidin micronization—through the reduction in hesperidin particle size—has been demonstrated to increase hesperidin bioavailability [21].

The hypothesis of the study was that bioavailability of 2S-hesperidin would be greater than the bioavailability of a mixture of 2S-/2R-hesperidin and that a micronization process would increase hesperidin bioavailability. Thus, the aim of the present study was to evaluate the effects of the micronization process and the 2R- and 2S-hesperidin diastereoisomers ratio on hesperidin bioavailability. To that end, we performed a nutritional intervention study to determine and compare the bioavailability and pharmacokinetics parameters of three sweet orange (*Citrus sinensis*)-derived hesperidin supplements formed by a 2S-diastereoisomer-enriched hesperidin and a racemic mixture of the 2R- and 2S- diastereoisomers, to which the micronization process had been applied or not.

## 2. Materials and Methods

### 2.1. Hesperidin Supplements

The three hesperidin supplements were Hesperidin epimeric mixture (HEM), micronized Hesperidin epimeric mixture (MHEM) and micronized 2S-Hesperidin (M2SH; Cardiose^®^) and consisted of two capsules with 250 mg of a natural sweet orange hesperidin extract and maltodextrin as a carrier. All three hesperidin extracts were supplied by HealthTech BioActives (HTBA, Murcia, Spain) with approximately 90% of the extract being hesperidin with different proportions of 2S-/2R- diastereoisomers, and the remaining 10% being hesperidin-related compounds, with different particle sizes (Table 1). The capsules with the hesperidin extracts were manufactured by the Mireia Valls pharmacy (Cambrils, Tarragona, Spain).

HEM is the conventional hesperidin obtained from sweet orange through alkaline extraction conditions, with part of the diastereoisomer 2S- transformed into the diastereoisomer 2R- due to extraction process. The particle size of the hesperidin extract obtained was approximately less than 100 µm for 90% of the particles and less than 10 µm for 10% of the particles.

MHEM was obtained as explained for HEM with the difference being that the extract was subjected to a micronization process so that 90% of the hesperidin particles were less than 10 µm in size.

M2SH is a natural extract of sweet orange that, due to its unique manufacturing process, contains a high content of the natural hesperidin diastereoisomer 2S-, and which has been subjected to a micronization process, with 90% of the particles of hesperidin having a size of less than 10 µm.

The three treatments were similar in appearance and were differentiated only by a code (HESP-01, HESP-02 and HESP-03) assigned by an independent researcher not related to the study to guarantee blinding.

### 2.2. Participants

Healthy subjects from the general population were recruited by means of news in the newspapers, tableaux advertisements and social networks at the Hospital Universitari Sant Joan (HUSJ)-Eurecat, Reus, Spain, between 12 June and 1 August 2019.

From 32 subjects assessed for eligibility, 30 individuals were recruited to carry out the first phase of the study, of which 15 participants carried out the postprandial study. Participants were men and women aged ≥18 years willing to provide informed consent before the initial screening visit. The exclusion criteria were: use of multivitamin supplements or supplements or phytotherapeutic products that interfere with the treatment under study up to 30 days before the start of the study; use of antibiotics up to 30 days before the start of the study; active smoking; present intolerances and/or food allergies related to hesperidin; subjects diagnosed with chronic gastrointestinal disease; the presence of some chronic diseases with clinical manifestation; being vegetarian; being pregnant or intending to become pregnant; being in the breastfeeding period; and current or past participation in a clinical trial or nutritional intervention study in the last 30 days prior to study enrolment.

Participants signed informed consent prior to their participation in the study, which was approved by the Clinical Research Ethical Committee of *Institut d’Investigació sanitaria Pere Virgili* (Ref. CEIM: 070/2019), Reus, Spain. The protocol and trial were conducted in accordance with the Helsinki Declaration and Good Clinical Practice Guidelines of the International Conference of Harmonization (GCP ICH) and reported as CONSORT criteria. The trial was registered at Clinical-Trials.gov: NCT03984916.

### 2.3. Study Design

The study consisted of two phases. In the first phase, 30 volunteers consumed 500 mL of orange juice (OJ) with 345 mg of hesperidin [9], and the levels of hesperidin excreted in urine were determined. The OJ used in the first phase of the study was the hesperidin OJ used in previous studies conducted by our group [9]. The objective of this first phase of the study was to select volunteers with intermediate hesperidin metabolite levels excreted in urine to reduce the variability in the results in the second phase of the study [21,23].

The second phase of the study was a randomized, crossover, postprandial, double-blind intervention study. The participants selected from the first phase of the study were randomly divided into 6 groups of sequences. The randomization plan was generated by using the website Randomization.com (http://www.randomization.com) accessed on 8 January 2020. Because all participants received all interventions (HEM, MHEM or M2SH), restrictions such as blocking were unnecessary. Participant assignment to each treatment was at a ratio of 1:1:1. Participants, researchers and the statistician remained blinded to the type of product administered in each study visit.

During the study, participants performed a total of five visits consisting in a pre-selection visit to check inclusion/exclusion criteria and—in the case of satisfying the eligibility criteria—a visit in the first phase of the study and—for the participants selected to perform the second phase of the study—three visits in the postprandial study for each of the hesperidin supplements.

**Figure 1:**
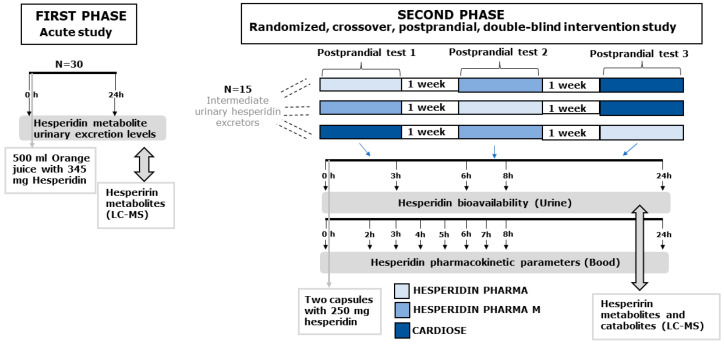
Schematical representation of the clinical study.

For the first phase of the study—after at least 8 h of fasting—the participants’ urine was collected before the intake of 500 mL of OJ (basal urine) and after the intake of the OJ for 24 h, accumulated in a single volume urine (Figure 1). Urine samples were homogenized and centrifuged at 1900 g for 10 min at 4 °C, and the supernatant was collected and subdivided into aliquots which were stored at −80 °C until analysis.

For the postprandial study, participants consumed each of the three hesperidin supplements in each study visit according to the randomization sequence. The washout period between each visit was one week. In each of the three visits, 10 mL of blood was drawn before consuming the hesperidin supplement (basal blood) and at the following time points after the consumption of the supplement: 2 h, 3 h, 4 h, 5 h, 6 h, 7 h, 8 h and 24 h (Figure 1). Serum was obtained from each blood sample by centrifugation at 1700× *g* for 15 min at 20 °C and subdivided into aliquots which were stored at −80 °C until analysis. Furthermore, the participant collected a fresh urine sample in the morning before consuming the corresponding hesperidin supplement (basal urine) an after consuming it over the time periods 0–3 h, 3–6 h, 6–8 h and 8–24 h, in urine collection flasks (Figure 1). The total volume of each urine fraction was recorded, and urine samples were processed as explained above.

Each of the hesperidin supplements was consumed by the study participants along with a light breakfast without flavonoid or phenolic compounds. No other food or drink except for water was allowed for the next 5 h. Five hours after consuming the hesperidin supplement, participants ate a light meal without flavonoid or phenolic compounds following recommendations made by nutritionists, based on 200 mL of vegetables broth, 42 g of white rice, 60 g of tuna canned in sunflower oil, 125 mL of whole-fat yogurt and water as a drink. Eight hours after consuming the hesperidin supplement, participants were instructed to go home and to continue following a diet low in flavonoids and phenolic compounds—by avoiding phenolic compound-rich fruits and vegetables, high-fibre products and beverages such as tea, coffee, fruit juices and wine—up to 24 h after consumption of the hesperidin supplement, where blood and urine samples were collected.

In order to homogenize the postprandial response in the bioavailability of hesperidin for the two days prior to each of the study visits, the first phase visit and each of the postprandial visits—and up to 24 h after having consumed the OJ or the hesperidin supplement—the participants were asked not to practice exercise or consume alcohol and to follow a diet low in flavonoids and phenolic compounds.

### 2.4. Outcomes

Relative urinary hesperidin excretion values (% of hesperidin ingested) obtained in the first phase of the study were used to select the participants for the second phase of the study. It was calculated by the ratio between the sum of hesperidin metabolites and the amount of hesperidin consumed by the participants (345 mg). With these values, 18 volunteers with intermediate relative urinary hesperidin excretion values were selected for the second phase.

The main outcome of the study was the hesperidin bioavailability determined by means of relative urinary hesperidin excretion (% of hesperidin ingested) considering the sum of hesperidin metabolites and catabolites.

The secondary outcomes were the relative urinary hesperidin excretion considering only hesperidin metabolites and considering only hesperidin catabolites; the area under the curve (AUC) of the serum sum of hesperidin metabolites and catabolites, of serum hesperidin metabolites and of serum hesperidin catabolites; the pharmacokinetic parameter time to reach peak concentration (T_max_); peak concentration (C_max_); and the elimination half-time (t _½_) of serum sum of hesperidin metabolites and catabolites, of serum hesperidin metabolites and of serum hesperidin catabolites.

To obtain the main and secondary outcomes, the urinary levels of hesperidin metabolites and catabolites at baseline—before consuming the hesperidin supplement—and up to 24 h after consuming the hesperidin supplement in four urine fractions (0–3 h; 3–6 h; 6–8 h; 8–24 h) were determined [19,29]. In addition, the serum levels of hesperidin metabolites and catabolites at baseline and at 2 h, 3 h, 4 h, 5 h, 6 h, 7 h, 8 h and 24 h after consuming the hesperidin supplement were quantified [19,24,29]. To obtain the relative urinary hesperidin excretion in the first phase of the study, the urinary levels of hesperidin metabolites before consuming the OJ and in the 24 h urine after consuming the hesperidin supplement were determined.

Descriptive outcomes including anthropometric parameters and blood pressure were measured as previously described by our group [30]. In addition, blood parameters—including serum levels of glucose, total cholesterol, HDL-cholesterol, LDL-cholesterol and triglycerides—were measured by standardized methods in a Cobas Mira Plus autoanalyzer (Roche Diagnosis Systems, Madrid, Spain).

### 2.5. Determination of Hesperidin Metabolites and Catabolites in Urine and Serum Samples

Hesperetin and related metabolites were determined in urine and serum samples by liquid chromatography coupled to triple quadrupole mass spectrometry (LC-QqQ) using a UHPLC 1290 Infinity II Series coupled to a QqQ/MS 6490 Series (Agilent Technologies, Palo Alto, CA, USA) and an ACQUITY UPLC BEH C18 (100 × 2.1 mm; 1.8 µm) column (Waters, Milford, MA, USA). Mobile phase A was 100% water with 0.5% acetic acid and 10 mM ammonium acetate, and mobile phase B was 100% methanol. The column temperature was set at 45 °C and the injection volume was 5 µL. The triple quadrupole operated in negative electrospray ionization mode (ESI-). The source conditions were set at 25 psi for the nebulizer gas, 240 °C for the gas temperature, 17 L/min for the gas flow, 250 °C for the sheath gas temperature, 12 L/min for the sheath gas flow, 3500 V for the capillary voltage and 0 V for the nozzle voltage. The standards used for quantification purposes were: Hesperetin and Hesperidin (Extrasynthese, Genay, France); Hesperetin 7-O-beta-D-glucuronide, Hesperetin 3-O-beta-D-glucuronide and Hesperetin 7,3′-di-O-beta-D-glucuronide (Toronto Research Chemicals; North York, ON, Canada); Hesperitin 7-O-sulfate (Santa Cruz Biotechnology, Dallas, TX, USA). Hesperitin-d3 and Diosmetin 7-O-β-D-Glucuronide-d3 (Toronto Research Chemicals; North York, ON, Canada) were used as internal standards.

Hesperidin catabolites were determined in urine and serum samples by liquid chromatography coupled to quadrupole time of flight mass spectrometry (LC-qTOF) using a UHPLC 1290 Infinity II Series coupled to a qTOF/MS 6550 Series (Agilent Technologies) and a Zorbax Eclipse C18 (150 × 2.1 mm; 1.8 µm) column (Agilent Technologies). Mobile phase A was 100% water with 0.2% acetic acid, and mobile phase B was 100% methanol. The column temperature was set at 45 °C, and the injection volume was 5 µL. Data acquisition was carried out in a full-scan over a mass-range of 50–1200 *m*/*z*, and the fragmentation studies were carried out at 10 V of collision energy. The source conditions were set at 25 psi for the nebulizer gas, 240 °C for the gas temperature, 17 L/min for the gas flow, 250 °C for the sheath gas temperature, 12 L/min for the sheath gas flow, 3000 V for the capillary voltage and 1500 V for the nozzle voltage. The standards used for quantification purposes were: 4′-Hydroxyphenylacetic acid, Hippuric acid, 4′-Hydroxycinnamic acid, 3-(4′-hydroxyphenyl)propanoic acid, 4′-Hydroxy-3′-methoxyphenylacetic acid, 3′-Hydroxy-4′-methoxyphenylacetic acid, 3′-hydroxyphenylacetic acid, 2-Hydroxy-2-(4′-hydroxy-3′-methoxyphenyl)acetic acid, 3′,4′-Dimethoxyphenylacetic acid, 3-Hydroxy-3-(3′-hydroxyphenyl)propanoic acid, 3,4-Dihydroxybenzoic acid, 3′,4′-Dihydroxyphenylacetic acid, 3-(3′,4′-dihydroxyphenyl)propanoic acid, 3-Hydroxy-4-methoxybenzoic acid, Phenylacetic acid and 3-Phenylpropionic acid (Sigma Aldrich; San Luis, MO, USA); 3-(3′-methoxy-4′-hydroxyphenyl)propanoic acid, 3-(4-hydroxy-3′-methoxyphenyl)propanoic acid-4′-sulfate, 2-Hydroxy-2-(4′-hydroxyphenyl)acetic acid, 3-(3′-hydroxy-4′-methoxyphenyl)propanoic acid, 4′-hydroxycinnamic acid 4-O-Glucuronide, 4′-Hydroxyhippuric acid, 3′-methoxycinnamic acid-4′-O-glucuronide, 3-(4-hydroxy 3′-methoxyphenyl)propanoic acid-4′-O-glucuronide and Caffeic acid-3′-O-glucuronide (Toronto Research Chemicals, Ontario, Canada). (+\−)-2-Hydroxy-2-(4′-hydroxy-3′-methoxyphenyl)acetic acid D3 (ring-D3) (Sigma Aldrich; San Luis, MO, USA); 3-Hydroxyhippuric Acid-^13^C_2_^15^N and Diosmetin 7-O-β-D-Glucuronide-d3 (Toronto Research Chemicals, ON, Canada); (4-Hydroxy-3-methoxyphenyl-d_3_) acetic-alpha,alpha-d2 acid and (4-Hydroxyphenyl-2,3,5,6-d4) acetic-2,2-d_2_ Acid (Cluzeau Info Labo, Sainte-Foy-la-Grande, France); and 3-(3-hydroxy-4-methoxyphenyl)propanoic acid-d_3_ (Santa Cruz Biotechnology; Dallas, TX, USA), were used as internal standards.

Urine samples were thawed at 4 °C. Fifty microliters of urine was mixed with 887.5 μL of water with 0.1% formic acid containing the internal standards (15 ng/mL of Hesperetin-d3, 120 ng/mL of Diosmetin 7-O-glucuronide-d3, 10 ng/mL of 2-Hydroxy-2-(4′-hydroxy-3′-methoxyphenyl)acetic acid -d3, (4-Hydroxy-3-methoxyphenyl-d3) acetic-alpha,alpha-d2 acid, (4-Hydroxyphenyl2,3,5,6-d4) acetic-2,2-d2 acid, 3-Hydroxyhippuric acid-13C2, 15N and 3-(3-hydroxy-4methoxyphenyl)propanoic acid-d3). Then, the mixture was vortexed and centrifuged for 5 min at 4 °C and 15,000 rpm. The supernatant was transferred to a glass vial for its analysis.

Serum samples were thawed at 4 °C. Then, 87.5 μL of serum was mixed with 525 μL of methanol containing the internal standard (400 ng/mL of Diosmetin 7-O-glucuronide-d_3_ and 20 ng/mL of Hesperetin-d_3_, 2-Hydroxy-2-(4′-hydroxy-3′-methoxyphenyl)acetic acid -d_3_, (4-Hydroxy-3-methoxyphenyl-d_3_) acetic-alpha,alpha-d_2_ acid, (4-Hydroxyphenyl-2,3,5,6-d_4_) acetic-2,2-d_2_ acid, 3Hydroxyhippuric acid-^13^C_2_^15^N and 3-(3-hydroxy-4-methoxyphenyl)propanoic acid-d_3_). Then, the mixture was vortexed and centrifuged for 5 min at 4 °C and 15,000 rpm. The supernatant was transferred to a new tube and evaporated to dryness in the SpeedVac at 45 °C. Samples were reconstituted with 100 μL of methanol: 1% formic acid in water (25:75, *v*/*v*) and transferred to a glass vial for its analysis.

The assignment of the metabolites and catabolites was performed by direct comparison with the commercial standards available or by bibliographic information using chromatographic behaviour, molecular ion ([M-H]-) and fragmentation patterns [16,18,22]. The obtained calibration curves were used for the quantification of their corresponding metabolites/catabolites. For the rest of compounds, the analysis was semi-quantitative. The behaviour of each compound was evaluated, and the calibration curve chosen to calculate its concentration was the one of the metabolites/catabolites that behaved more alike.

### 2.6. Sample Size

The sample size was calculated using the GPower 3.1 software, considering relative urinary hesperidin excretion values (% of hesperidin ingested) as the main variable. To detect differences between the hesperidin supplements (HEM, MHEM and M2SH) of 20%, a mean and a standard deviation of 4.9% and 1.8%, respectively [14,19,24,25,29,31], a power of 90% and a confidence level of 95%, the sample size was 13 participants.

Considering that most of the population presents an intermediate excretion level of hesperidin in urine [21,22], the number of individuals considered to carry out the first phase of the study was 30.

### 2.7. Statistical Analysis

The parametricity of the variables was examined by Kolmogorov–Smirnov tests, analyses of skewness and kurtosis were performed, and logarithmic transformation was performed if required. Wilcoxon tests or general linear models for repeated measures with Bonferroni correction were performed for assessing the time changes in hesperidin metabolites and phenolic catabolites in urine and serum. Comparison between formulations was assessed by Student’s t test or Wilcoxon tests for related samples. When plasma concentrations were lower than the low limit of quantification of the analytical method, half of the detection limit was used as a value. Missing values were imputed by linear regression analyses. Observed plasma pharmacokinetic parameters were directly extracted from plasma concentrations over time curve: maximum peak concentrations (C_max_), time to reach peak concentrations (T_max_), half-life elimination (t _½_) and areas under the concentrations–time curves between 0 h and 24 h (AUC_0–24h_). AUCs were calculated using the linear trapezoidal rule.

The level of statistical significance was set at *p* ˂ 0.05. Data were analyzed using the SPSS software version 26. 

## 3. Results

### 3.1. Study Population

**Figure 2:**
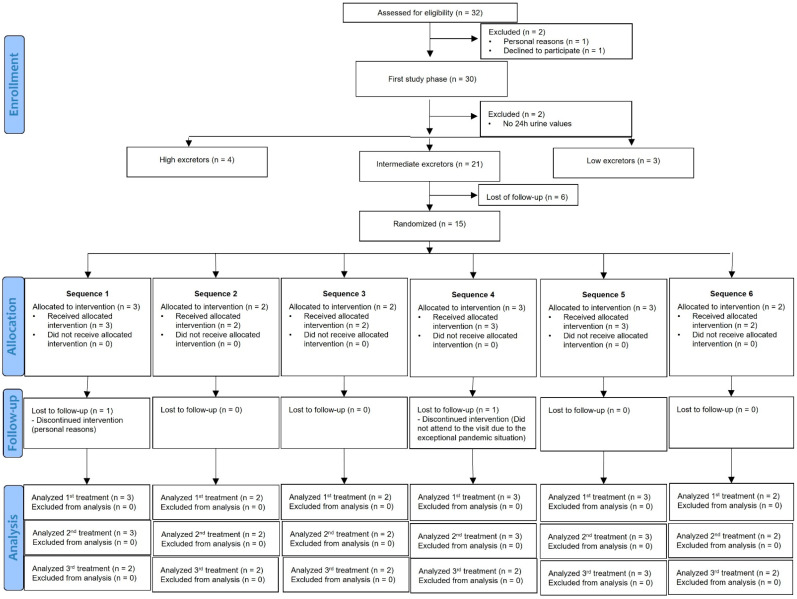
Participant flowchart. Sequence 1: Hesperidin epimeric mixture (HEM), micronized Hesperidin epimeric mixture (MHEM), micronized 2S-Hesperidin (M2SH); Sequence 2: HEM, M2SH, MHEM; Sequence 3: MHEM, HEM, M2SH; Sequence 4: MHEM, M2SH, HEM; Sequence 5: M2SH, HEM, MHEM; Sequence 6: M2SH, MHEM, HEM.

From the 32 eligible participants, 30 were included for the first phase of the study and, from these, 2 participants were excluded because no 24 h urine values were available. From the 28 participants, 21 were intermediate excretors of hesperidin and 6 participants declined to take part in the second phase of the study. Therefore, 15 participants were randomized for the second phase of the study. Of these 15 participants, 1 participant presented problems with blood extraction during the second postprandial study, obtaining samples for the first postprandial and up to 6 h after consumption of the hesperidin supplement for the second postprandial. Another participant only completed two postprandial studies due to the restrictions caused by the SARS-CoV-2 pandemic. Finally, data from 14 participants in the HEM group, 14 participants in the MHEM group and 14 participants in the M2SH group were available. A CONSORT flowchart of the study is described in Figure 2.

The baseline characteristics from the 30 participants included in the study are described in Supplementary Table S1. Mean relative urinary hesperidin excretion determined as hesperetin or hesperetin conjugates in urine was 2.67 ± 1.79%. The range of relative hesperidin excretion levels was 0.64–8.07%, and participants could be classified into low, intermediate and high urinary hesperidin excretors with values of relative urinary hesperidin excretion of 0.74 ± 0.11%, 2.31 ± 1.01% and 6.06 ± 1.46%, respectively (Supplementary Table S1). These results are similar to those obtained in other studies [14,18,19,20,24,29].

### 3.2. Hesperidin Bioavailability: Relative Urinary Hesperidin Excretion

Supplementary Tables S2–S4 show the quantities of hesperidin metabolites and catabolites in urine before and after the intake of each of the three hesperidin supplements. A portion of the catabolites detected and quantified in urine were non-specific hesperidin catabolites that were also products of endogenous pathways unrelated to hesperidin supplements intake and, therefore, could interfere in the results. Therefore, the analysis of relative urinary hesperidin excretion was carried out considering only the urinary hesperidin catabolites that could be specific of hesperidin metabolism on the basis of the data described by Pereira-Cano et al. [16,22,27] and also those catabolites that showed a similar pattern to 3-(3′-hydroxy-4′-methoxyphenyl)propionic acid (HMPPA), as it has been described as a specific hesperidin catabolite [18].

The quantity of the sum of hesperidin metabolites and hesperidin catabolites increased linearly from baseline to 8–24 h in all treatments. The 0–24 h urinary excretion of hesperidin metabolites and catabolites was significantly higher after M2SH supplement intake than after the intake of the other two hesperidin supplements (Table 2). Therefore, the overall amount of hesperidin excreted in urine, the relative urinary hesperidin excretion and the bioavailability were significantly higher for M2SH than for the other two hesperidin supplements.

Concerning hesperidin metabolites, relative urinary hesperidin excretion values observed for the three hesperidin supplements are in accordance with the results reported by other authors considering only hesperidin metabolites for hesperidin bioavailability quantification [18,19,29,31,32]. Urinary hesperidin metabolites quantities also increased linearly from baseline to 8–24 h in all treatments (*p* ˂ 0.005). The higher 0–24 h urinary hesperidin metabolites excretion, however, was observed after MHEM intake, which was significantly higher than after the other two treatments. Furthermore, the lower relative urinary hesperidin excretion was observed after M2SH intake, which was significantly lower than MHEM treatment and showed a tendency to be lower than HEM treatment (Table 2).

The quantity of hesperidin catabolites also increased linearly from baseline to 8–24 h in all treatments. In this case, the overall amount of hesperidin excreted in urine and therefore the bioavailability was significantly higher after M2SH supplement intake than after the intake of the other two hesperidin supplements (Table 2).

### 3.3. AUC and Pharmacokinetic Parameters

Tables S5–S7 show the concentration of hesperidin metabolites and catabolites in serum before and after the intake of each of the three hesperidin supplements. For the same reasons discussed above for the urine values, AUCs and pharmacokinetic parameters were calculated considering only the serum hesperidin catabolites that could be specific of hesperidin metabolism [16,18,22,27].

**Figure 3:**
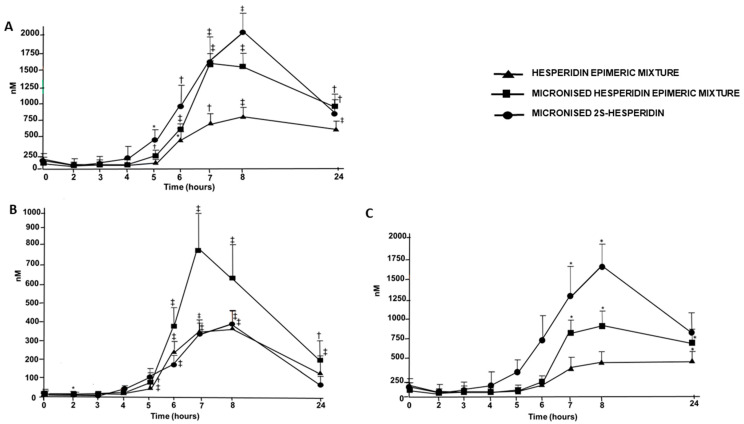
Time–response curve of the sum of serum hesperidin metabolites and catabolites (**A**), serum hesperidin metabolites (**B**) and serum hesperidin catabolites (**C**), after Hesperidin epimeric mixture (HEM), micronized Hesperidin epimeric mixture (MHEM) and micronized 2S-Hesperidin (M2SH) treatments. * *p* < 0.05, ^†^ *p* < 0.01, ^‡^ *p* < 0.001 versus baseline.

Table S8 and Figure 3A show the time–response curves of the sum of serum hesperidin catabolites and metabolites for the three hesperidin supplements. Curves were similar for the three hesperidin supplements, reaching the maximal values between 7 and 8 h. Higher concentrations were observed after M2SH and MHEM treatments. Table 3 shows the pharmacokinetic parameters obtained. C_max_ and AUC_0–24h_ were higher after M2SH and MHEM intake than after HEM intake, showing the M2SH supplement led to higher values in the percentage of change compared to the HEM supplement than the MHEM supplement. Moreover, AUC_0–24h_ for the M2SH supplement showed a tendency to be higher than that for the MHEM supplement. T_max_ was lower after M2SH intake compared to after HEM intake with a borderline significance.

The time–response curves of serum hesperidin metabolites’ maximal values were shown 7–8 h, as reported by other authors [14,25]. Hesperidin metabolites began to appear in serum approximately between 4 h and 5 h after hesperidin supplement consumption. These results indicate that hesperidin absorption occurs mainly in the colon, as has been previously described [18]. Curves were similar for the three hesperidin supplements, but higher concentrations were obtained after MHEM intake (Table S9 and Figure 3B). C_max_ and AUC_0–24h_ were higher after MHEM intake than after the intake of the other two hesperidin supplements. Values of T_max_ and t _½_ were similar in all groups (Table 4).

For hesperidin catabolites, the time–response curves after the ingestion of the three hesperidin supplements showed that the maximum values were reached between 8 and 24 h. Curves were similar for the three hesperidin supplements, but higher concentrations were obtained after M2SH intake (Table S10 and Figure 3C). AUC_0–24h_ was higher after M2SH intake than after the intake of the other two hesperidin supplements, whereas no significant differences were observed between MHEM and HEM supplements. C_max_ was higher after M2SH intake and MHEM intake than after HEM intake. Furthermore, C_max_ for the M2SH supplement was higher than that for the MHEM supplement although with borderline significance. Values of T_max_ were similar in all groups (Table 5).

## 4. Discussion

Results from several clinical trials evaluating the bioavailability of hesperidin from hesperidin extracts [33,34] and from natural sources [18,19,22,24,29,31] show a low bioavailability for hesperidin that may be due to various factors, including the predominance of 2R-hesperidin diastereoisomer in the hesperidin extracts, the low water solubility of hesperidin and the limited amount of hesperidin metabolites considered for hesperidin bioavailability quantification. In this study, the effect of stereochemistry and particle size on hesperidin bioavailability was evaluated by comparing the bioavailability of three hesperidin supplements with different proportions of the diastereoisomers 2S-/2R- and different particle size. Furthermore, both hesperidin metabolites and hesperidin catabolites were analysed and quantified in urine and serum samples after consumption of each of the three supplements to determine hesperidin bioavailability.

In order to exert its health effects, hesperidin must be bioavailable and absorbed from the gastrointestinal tract into the circulatory system. In our study, serum concentrations of hesperidin metabolites over time were in accordance with hesperidin metabolization in the colon [19]. It has been described that an amount of consumed hesperidin by humans is absorbed in the small intestine. However, the 70% of ingested hesperidin reaches the large intestine, where it must be hydrolyzed by microbial α-rhamnosidase activity in the colon into hesperetin aglycone prior to its absorption. Once absorbed, hesperetin can be conjugated into glucuronidated and sulfonated metabolites [22,35,36]. Hesperetin released through colonic bacteria can be further subjected to ring fission by the resident microbiota and broken down to yield a family of low-molecular-weight phenolic catabolites, which could be additionally metabolized before entering the systemic circulation [16,36,37]. According to the results reported by other authors [14,18,19,20,22,24,27,29], we observed a low hesperidin bioavailability when only hesperidin metabolites were considered, with relative hesperidin urinary recoveries between 5.2% and 9.2%. When hesperidin metabolites and catabolites were considered, the bioavailability of hesperidin increased markedly for the three hesperidin supplements, with relative hesperidin urinary recoveries between 43% and 70%. Overall, our results reinforce the idea that intestinal microbiota have a crucial role in the metabolism and absorption of hesperidin, including the production of metabolites and also catabolites of this flavanone [36,37,38].

Although the 2S-diastereoisomer of hesperidin is dominant in nature, current commercial hesperidin extracts are available as a mixture of both diastereoisomers due to the transformation of the 2S-diastereoisomer to the 2R-diastereoisomer during the industrial extraction process [11]. Interestingly, the results of our study show that the bioavailability of M2SH supplement—formed mainly by 2S-diastereoisomer and therefore a supplement with a hesperidin form closer to the natural hesperidin—was higher than the bioavailability of MHEM and HEM, which consist of a mixture of both hesperidin diastereoisomers, with a relative urinary hesperidin excretion of 70%, 55% and 43%, respectively. Furthermore, it is important to highlight that the higher bioavailability of hesperidin observed for the M2SH supplement was due to the greater formation of hesperidin catabolites compared to the other two hesperidin supplements. On the other hand, relative urinary excretion of hesperidin metabolites was higher for MHEM and HEM supplements than for the M2SH supplement. Serum results obtained in the study are in accordance with the results obtained in urine, with the M2SH supplement being the one that showed the higher AUC_0–24h_ and C_max_ values when considering hesperidin metabolites and catabolites or only hesperidin catabolites, whereas when only hesperidin metabolites were considered, the supplement with the higher AUC_0–24h_ and C_max_ was MHEM. These results are in accordance with other studies showing that the two hesperidin diastereoisomers may display distinct kinetic and bioavailability properties [11] and support the importance of considering the chirality of hesperidin in studies evaluating the bioavailability of this flavonoid. In this sense, in an in vivo study, after intravenous administration of racemic hesperetin to rats, R-diastereoisomer showed a significant 3.2-fold higher AUC compared to S-diastereoisomer [12]. In that study, catabolites were not considered for hesperidin absorption measurements. Our results support the hypothesis that 2S- and 2R-diastereoisomers of hesperidin present a differential metabolism by colonic microbiota. It has been demonstrated that intestinal microbiota are a key factor in the metabolism and absorption of hesperidin. However, the research has been mainly centred on the α-L-rhamnosidase bacterial activity, studying the ability of intestinal microbiota to hydrolyze hesperidin and establishing the species responsible for this activity, such as *Bifidobacterium catenulatum* and *Bifidobacterium pseudocatenultum* [39]. However, these studies did not consider the influence of hesperidin stereochemistry in microbiota metabolism. On the other hand, it is known that flavonoids can influence the quantity and quality of the intestinal microbiota in the colon and indirectly influence their own metabolism and bioavailability [37], although no studies have been carried out evaluating the influence of flavonoids’ stereochemistry on these processes.

The results obtained in our study demonstrate the importance of considering hesperidin catabolites not only in bioavailability studies, but also in studies evaluating the biological effects of this flavonoid, for several reasons. Firstly, and according to the results obtained in our study and also by other authors [18,26,27], hesperidin catabolites are the major contributors of hesperidin bioavailability values. Secondly, if only hesperidin metabolites are considered, it is likely that the results obtained in studies comparing the hesperidin bioavailability of different natural sources or hesperidin extracts may not be completely conclusive. Finally, the results obtained in this study suggest that the three hesperidin supplements M2SH, MHEM and HEM may present different bioactive effects due to their different bioavailability, related to a different contribution of the metabolism by the intestinal microbiota. Although the three hesperidin supplements share the same metabolites and catabolites, they differ in the contribution of these metabolites and catabolites to the overall amount of absorbed compounds. Recently, much attention has been paid to the bioavailability and physiological actions of polyphenol catabolites due to the high absorption level and, therefore, the potential physiological actions of these compounds [36]. For instance, urolithins—which are catabolites of ellagitannin—show higher antioxidant, anti-inflammatory and anti-proliferation activities than ellagitannin and ellagic acid [40,41,42]; 3,4-dihydroxybenzoic (protocatechuic) acid (PCA), which is a catabolite derived from the metabolism of several polyphenols such as procyanidins and various anthocyanins, exerts a wide range of biological effects, such as antioxidant, anti-inflammatory, anticarcinogenic and neuroprotective activities [37]; 3,4-dihydroxyphenylacetic acid (DOPAC) is one of the active phenolic acids derived from quercetin glycosides’ intestinal microbiota catabolism for which several activities have been demonstrated—including free radical scavenging and anti-inflammatory, antioxidant and anticancer properties [37]. Along with the potential beneficial effects of 2S-hesperidin, and its catabolites produced by colonic microbiota, in a parallel, randomized placebo-controlled trial with forty amateur cyclists, the consumption of 500 mg/day of CARDIOSE hesperidin extract for eight weeks increased the functional threshold power and maximum power [43] and also decreased fat mass and increased muscle mass [44] compared to the placebo group.

The results of the study show that the bioavailability of hesperidin—considering mainly the hesperidin metabolites—from the MHEM supplement was clearly higher than the hesperidin bioavailability from the HEM supplement. Considering that urinary data are indicative of the minimum absorbed fraction [45], the increase in absorption was 40% between both hesperidin supplements. The only difference between these two hesperidin supplements was the particle size of hesperidin, which is smaller in the MHEM supplement due to the application of the micronization process to the hesperidin extract. Several factors limit the bioavailability of hesperidin after oral intake, one of them being its poor water solubility [25,46], and micronization is a process that has been used to increase the bioavailability of hesperidin [21] and other polyphenols [45,47] by increasing its dissolution rate and therefore to improve the overall absorption through the reduction in particle size [45]. Therefore, our study reinforces the utilization of the micronization process to increase hesperidin bioavailability in hesperidin-derived products to enhance the biological effects of this flavonoid.

## 5. Conclusions

In conclusion, the results of this study demonstrate that the bioavailability of the 2S-hesperidin extract was higher compared to the standard mixture of 2S-/2R-hesperidin extract in healthy individuals due to a greater formation of hesperidin catabolites. Furthermore, the results obtained reinforce the use of micronization to increase hesperidin bioavailability. Considering the large differences in hesperidin metabolites and catabolites formed after M2SH, MHEM and HEM supplement ingestion, further studies are needed to evaluate potential differences in biological effects between hesperidin supplements with different proportions of 2S-/2R-diastereoisomers.

## Figures and Tables

**Table 1:** Products of the clinical study.

Product	Amount (mg)	-S/-R (%)	Particle ˂ 10 µm (%)	Hesperidin (%)	Water (%)	Hesperidin (mg)
HEM	500	56/44	10	94.4	2.10	462.1
MHEM	500	55/45	90	94.10	1.80	462.0
M2SH	500	93/7	90	92.60	2.50	451.4

HEM: Hesperidin epimeric mixture; MHEM: micronized Hesperidin epimeric mixture; M2SH: micronized 2S-Hesperidin.

**Table 2:** Quantities of Hesperidin metabolites and Hesperidin catabolites in urine before and after Hesperidin epimeric mixture (HEM), micronized Hesperidin epimeric mixture (MHEM) and micronized 2S-Hesperidin (M2SH) intake.

			Change from Baseline					
Variable /Intervention	Baseline (0 h)	0–3 h	3–6 h	6–8 h	8–24 h	*p* for Linear Trend	Total Change ^a^ (0 h Post-24 h)	Total Relative to Intake (%)
***Total metabolites ^b^, µmol***								
HEM (*n* = 13)	0.035 (0.01)	**0.080 (0.005)** *	**0.613 (0.22)** ^†^	**7.04 (2.43)** ^†^	**31.35 (5.15)** ^†^	**˂0.001**	39.1 (6.1)	5.2 (0.81)
MHEM (*n* = 14)	0.022 (0.003)	**0.111 (0.01)** ^†^	**0.691 (0.23)** ^†^	**17.05 (5.13)** ^†^	**49.84 (9.75)** ^†^	**˂0.001**	**68.5 (13)** ^‡^	**9.2 (1.6)** ^‡^
M2SH (*n* = 14)	0.077 (0.05)	**0.130 (0.03)** *	**1.03 (0.34)** ^†^	**5.08 (1.36)** ^†^	**20.25 (6.16)** ^†^	**0.005**	**24.9 (7.8)** ^‡ ≠^	**3.6 (1.0)** ^§ ≠^
***Total specific catabolites ^c^, µmol***								
HEM (*n* = 13)	23 (8.6)	31 (15)	**8.6(2.8)** *	29 (17)	**205 (33)** ^†^	**˂0.001**	284 (61)	37 (8.0)
MHEM (*n* = 14)	25 (16)	**40 (27)** *	37 (28)	**39 (15)** *	**230 (47)** ^†^	**˂0.001**	346 (114)	46 (15)
M2SH (*n* = 14)	27 (18)	**37 (19)** *	***36 (21)***	**67 (29)** ^†^	**339 (43)** ^†^	**˂0.001**	**488 (107)** ^¥ ≠^	**66 (14)** ^¥ ≠^
***Total metabolites plus specific catabolites, µmol***								
HEM (*n* = 13)	23 (8.6)	31 (15)	**9.2 (2.9)** *	36 (19)	**237 (32)** ^†^	**˂0.001**	326 (60)	43 (8.0)
MHEM (*n* = 14)	25(16)	**40 (27)** *	37 (28)	**56 (17)** *	**280 (47)** ^†^	**˂0.001**	414 (113)	55 (15)
M2SH (*n* = 14)	27 (18)	**38 (19)** *	***37 (21)***	**72 (29)** ^†^	**360 (42)** ^†^	**˂0.001**	**515 (106)** ^‡^	**70 (14)** ^‡ ≠^

Data expressed as mean (standard error). One participant had only a few urine data, making data imputation impossible. ^a^ Intertreatment comparisons (total change minus baseline). ^b^ Total urinary metabolites = Hesperetin (Hesp), 7, 3′ di-O-glucuronide (gluc)+ Hesp 7-O-gluc+ Hesp 3-O-gluc+ Hesp 7-O-sulfate+ Hesp. ^c^ Total urinary specific catabolites = 4′-Methoxycinnamic acid-3′ -glucuronide + 3-(3′- hydroxy- 4′-methoxyphenyl) propanoic acid + 3-(3′- hydroxy 4′-methoxyphenyl) propanoic acid-3′-sulfate+ 3-(3′- hydroxy 4′-methoxyphenyl) propanoic ac-id-3′-O-glucuronide + 3′-(3′-hydroxyphenyl) propanoic acid-4′-sulfate + 3′-(4′-hydroxyphenyl) propanoic acid-3′-sulfate + 3′-(3′-hydroxyphenyl) propanoic acid-4′-O-glucuronide + 3′-(4′-hydroxyphenyl) propanoic acid-3-O-glucuronide + 3′-Hydroxy-3-(3′-hydroxyphenyl) propanoic acid + 3′-Hydroxy-3-(3′-hydroxy-4′-methoxyphenyl)propanoic acid. Intratreatment comparison by Wilcoxon test. * *p* < 0.05, ^†^ *p* < 0.005 versus its baseline. Significant results in **bold** and borderline ones (*p* > 0.05 and <0.1) in ***italic bold***. Intertreatment comparisons (total change minus baseline) by Wilcoxon test: **^§^** *p* = 0.055, ^‡^ *p* < 0.05, ^¥^ *p* < 0.01 versus HEM treatment; ^≠^ *p* < 0.05 versus MHEM treatment.

**Table 3:** Non-compartmental single-dose kinetics of Hesperidin metabolites plus Hesperidin catabolites after Hesperidin epimeric mixture (HEM), micronized Hesperidin epimeric mixture (MHEM) and micronized 2S-Hesperidin (M2SH) treatments.

Parameter	Treatment			*p* for Treatments			% of Change *		
	HEM (*n* = 13)	MHEM (*n* = 13)	M2SH (*n* = 13)	MHEM vs. HEM	M2SH vs. HEM	M2SH vs. MHEM	MHEM vs. HEM	M2SH vs. HEM	M2SH vs. MHEM
Dose, mg	462.1	462	451.4						
AUC_0–24_ (nmol.h^−1^)	13,510 ± 4638 ^a^ 13,680 (11,294; 17,294)	23,114 ± 11,579 ^a^ 25,288 (10,497; 34,502)	28,096 ± 13,344 ^a^ 21,775 (19,653; 39,712)	**0.012** ^†^	**<0.001** ^†^	***0.084***	74 ± 83 ^a^ 58 (2.5; 138)	118 ± 99 ^a^ 77 (43; 244)	40 ± 67 ^a^ 24 (−13; 80)
C_max,_ nmol	1022 ± 366 ^a^ 971 (751; 1288)	2296 ± 1190 ^a^ 2478 (949; 3433)	2497 ± 1373 ^a^ 2181 (891;3412)	**0.001** ^†^	**<0.001** ^†^	0.489	139 ± 118 ^a^ 133 (48; 198)	163 ± 119 ^a^ 140 (66; 252)	37 ± 95 ^a^ −3.6 (−32; 102)
T_max_, h	15.0 ± 8.7 ^a^ 8.0 (7.0; 24)	12.7 ± 7.8 ^a^ 8.0 (7.5; 24)	10.4 ± 6.0 ^a^ 8.0 (8; 8)	0.233	***0.079***	0.231	−8.7 ± 28 ^a^ 0.00 (0.00; 0.00)	−17.5 ± 37 ^a^ 0.00 (−67; 14)	−8.0 ± 26 ^a^ 0.00 (0.00; 0.00)
t _½_ (h) ^b^		14.5 ± 5.3 ^a^ 14.6 (9.8; 19)	NC	NC	_	_	_	_	_

Data expressed as mean ± standard deviation and ^a^ median (25–75th). * % of Change, percentage of change. AUC_0–24_, area under the curve 0–24 h. C_max_, plasma maximal concentration; T_max,_, time to maximal concentration; t _½_, elimination half-life, ^b^ data from only 5 individuals undergoing HEM treatment. NC, non-computable, data could not be calculated because C_max_ was at 8 h or 24 h in the most part of the individuals. Student’s t test for related samples or Wilcoxon test. ^†^ After logarithmic transformation of the data. Significant results in **bold** and borderlines ones in ***italic bold***.

**Table 4:** Non-compartmental single-dose kinetics of Hesperidin metabolites after Hesperidin epimeric mixture (HEM), micronized Hesperidin epimeric mixture (MHEM) and micronized 2S-Hesperidin (M2SH) treatments.

Parameter	Treatment			*p* for Treatments			% of Change *		
	HEM (*n* = 14)	MHEM (*n* = 14)	M2SH (*n* = 14)	MHEM vs. HEM	M2SH vs. HEM	M2SH vs. MHEM	MHEM vs. HEM	M2SH vs. HEM	M2SH vs. MHEM
Dose, ***mg***	462.1	462	451.4						
AUC_0–24_ (nmol.h^−1^)	4730 ± 3333 ^a^ 3993 (2547; 5602)	8264 ± 5722 ^a^ 7462 (3773; 13,509)	4550 ± 3941 ^a^ 3137 (2192; 5494)	**0.024**	0.644	**0.013**	87 ± 139 ^a^ 80 (−23; 141)	−9.5 ± 83 ^a^ −24 (−56; 72)	−41 ± 281 ^a^ −33 (−73; 29)
C_max_, nmol	406 ± 229 ^a^ 392 (229; 562)	971 ± 722 ^a^ 911 (323; 1529)	458 ± 325 ^a^ 358 (237; 527)	**0.006** ^†^	0.775	**0.007** ^†^	146 ± 174 ^a^ 146 (−5.2; 186)	28.8 ± 106 ^a^ −13.7 (−45; 87)	−22.6 ± 61 ^a^ −54 (−74; 36)
T_max_, h	10.8 ± 7.1 ^a^ 7.5 (7; 12)	9.7 ± 6.1 ^a^ 8.0 (7; 8.0)	8.6 ± 4.5 ^a^ 8.0 (7; 8.0)	1.00	0.347	0.349	10.7 ± 60.1 ^a^ 0.00 (−7; 14)	−4.6 ± 23.2 ^a^ 0.00 (−6; 0.00)	−3.9 ± 22.7 ^a^ 0.00 (−12; 14)
t _½_ (h) ^b^	4.85 ± 3.7 ^a^ 3.3 (2.7; 5.5)	6.02 ± 5.6 ^a^ 3.7 (2.4; 12)	4.62 ± 5.5 ^a^ 2.4 (2.2; 5.9)	0.465	0.345	0.655	2.67 ± 15.8 ^b^ 2.53 (−12; 18)	−18.1 ± 27.9 ^b^ −22.2 (−39; 5)	−0.16 ± 16.0 ^b^ −0.16 (−11; 11)

Data expressed as mean ± standard deviation and ^a^ median (25–75th). * % of Change, percentage of change. AUC_0–24_, area under the curve 0–24 h. C_max_, plasma maximal concentration; T_max,_, time to maximal concentration; t _½_, elimination half-life, ^b^ data from only 5 individuals undergoing HEM and M2SH treatments, and 4 individuals undergoing MHEM treatment. In the rest of the cases, data could not be calculated because C_max_ was at 8 h or 24 h, or because 24 h data were greater than those at 8 h. Student’s t test for related samples or Wilcoxon test. ^†^ After logarithmic transformation of the data. Significant results in **bold.**

**Table 5:** Non-compartmental single-dose kinetics of Hesperidine catabolites after Hesperidin epimeric mixture (HEM), micronized Hesperidin epimeric mixture (MHEM) and micronized 2S-Hesperidin (M2SH) treatments.

Parameter	Treatment			*p* for Treatments			% of Change *		
	HEM (*n* = 13)	MHEM (*n* = 13)	M2SH (*n* = 13)	MHEM vs. HEM	M2SH vs. HEM	M2SH vs. MHEM	MHEM vs. HEM	M2SH vs. HEM	M2SH vs. MHEM
Dose, mg	462.1	462	451.4						
AUC_0–24_ (nmol.h^−1^)	8438 ± 3583 ^a^ 8511 (6171; 10,010)	14,596 ± 9445 ^a^ 15,095 (7522; 22,322)	23,674 ± 4332 ^a^ 18,104 (11,404; 36,270)	0.117 ^†^	**<0.001** ^†^	**0.004** ^†^	82 ± 97 ^a^ 94 (5.8; 172)	183 ± 139 ^a^ 144 (60; 288)	103 ± 126 ^a^ 45 (20; 154)
C_max,_ nmol	674 ± 270 ^a^ 670 (500; 843)	1364 ± 846 ^a^ 1240 (818; 1982)	2085 ± 1344 ^a^ 1840 (891; 3111)	**0.007**	**0.002**	***0.075***	113 ± 103 ^a^ 94 (39; 196)	220 ± 171 ^a^ 196 (78; 337)	136 ± 319 ^a^ 29 (−4; 133)
T_max_, h	15.1 ± 8.6 ^a^ 8.0 (7.0; 24)	12.3 ± 7.7 ^a^ 8.0 (7.7; 24)	12.8 ± 4.5 ^a^ 8.0 (8; 24)	0.111	0.665	0.581	−15.1 ± 31 ^a^ 0.00 (−46; 0.00)	3.6 ± 70 ^a^ 0.00 (−50; 14)	13.9 ± 60 ^a^ 0.00 (0.00; 7.1)
t _½_ (h) ^b^	47 ± 34 ^a^ 39 (17; 86)	NC	NC	_	_	_	_	_	_

Data expressed as mean ± standard deviation and ^a^ median (25–75th). * % of Change, percentage of change. AUC_0–24_, area under the curve 0–24 h. C_max_, plasma maximal concentration; T_max_, time to maximal concentration; t _½_, elimination half-life, ^b^ data from only 5 individuals undergoing HEM treatment. NC, non-computable, data could not be calculated because C_max_ was at 8 h or 24 h. Student’s t test for related samples or Wilcoxon test. ^†^ After logarithmic transformation of the data. Significant results in **bold** and borderlines ones in ***italic bold***.

## Data Availability

The data presented in this study are available on request from the corresponding author.

## Supplementary Materials

The following supporting information can be downloaded at: https://www.mdpi.com/article/10.3390/nu14122481/s1. Table S1: Baseline characteristics and relative urinary hesperidin values of participants in the first phase of the study; Table S2: Quantities of Hesperidin metabolites and Hesperidin catabolites in urine before and after Hesperidin epimeric mixture (HEM) ingestion; Table S3: Quantities of Hesperidin metabolites and Hesperidin catabolites in urine before and after micronized Hesperidin epimeric mixture (MHEM) ingestion; Table S4: Quantities of Hesperidin metabolites and catabolites in urine before and after micronized 2S-Hesperidin (M2SH) ingestion; Table S5: Concentration of Hesperidin metabolites and catabolites in serum before and after Hesperidin epimeric mixture (HEM) ingestion; Table S6: Concentration of Hesperidin metabolites and catabolites in serum before and after micronized Hesperidin epimeric mixture (MHEM) ingestion; Table S7: Concentration of Hesperidin metabolites and catabolites in serum before and after micronized 2S-Hesperidin (M2SH) ingestion; Table S8: Concentration of Hesperidin metabolites plus Hesperidin catabolites (nM) in serum before and after Hesperidin epimeric mixture (HEM), micronized Hesperidin epimeric mixture (MHEM) and micronized 2S-Hesperidin (M2SH) treatments; Table S9: Concentration of Hesperidin metabolites (nM) in serum before and after Hesperidin epimeric mixture (HEM), micronized Hesperidin epimeric mixture (MHEM) and micronized 2S-Hesperidin (M2SH) treatments. Table S10: Concentration of Hesperidin catabolites (nM) in serum before and after Hesperidin epimeric mixture (HEM), micronized Hesperidin epimeric mixture (MHEM) and micronized 2S-Hesperidin (M2SH) treatments.
