# Supplementary material for: Hesperidin Bioavailability Is Increased by the Presence of 2S-Diastereoisomer and Micronization—A Randomized, Crossover and Double-Blind Clinical Trial
Source: Nutrients. 2022 Jun 15;14(12):2481. doi: 10.3390/nu14122481 (PMC9231284; doi:10.3390/nu14122481)
Supplement: Supplementary file 1 [file nutrients-14-02481-s001.zip › nutrients-1736017-supplementary.pdf]

Table S1. Baseline characteristics and relative urinary hesperidin values of participants in the first phase of the study.

Variable	Mean ± SD
Age, *years*	41.9 ± 12.4
Females, *%*	43.8%
Systolic blood pressure, *mm Hg*	121 ± 12.4
Diastolic blood pressure, *mm Hg*	75 ± 10.2
Weight, kg	75.5 ± 14.7
Body mass index, kg/m^2	26.1 ± 4.08
Waist circumference, *cm*	89.3 ± 13.7
Glucose, *mg/dL*	92.6 ± 11.2
Cholesterol, *mg/dL*	
Total	184 ± 43.9
LDL	110 ± 35.9
HDL	59.7 ± 15.6
Triglycerides, *mg/dL*	82.1 ± 40.7
Hemoglobin, *g/dL*	14.5 ± 0.951
Creatinine, *mg/dL*	0.919 ± 0.191
Total bilirubin, *mg/dL*	0.719 ± 0.740
Aspartate aminotransferase, *UI/L*	20 ± 4.24
Alanine aminotransferase, *UI/L*	21 ± 10
Gamma glutamyl transferase, *UI/L*	18.1 ± 11.0
Alkaline phosphatase, *UI/L*	64.3 ± 18.7
Thyrotropin, mUI/mL	1.89 ± 0.938
Hesperidin excretion (%) (N=28)	2.67 ± 1.79
High excretors (N=4)	6.06 ± 1.46
Intermediate excretors (%; N=21)	2.31 ± 1.01
Low excretors (%; N=3)	0.743 ± 0.112

LDL, low density lipoproteins; HDL, high density lipoproteins. N=30.

Table S2. Quantities of Hesperidin metabolites and Hesperidin catabolites in urine before and after Hesperidin epimeric mixture (HEM) ingestion.

Variable	0 h	0-3 h	3-6 h	6-8 h	8-24 h
Metabolites, µmol					
Hesperetin 7,3'-di-O-glucuronide	0.001 ± 0.001	0.001 ± 0.001	0.046 ± 0.021	0.41 ± 0.11	2.79 ± 0.51
Naringenin 7-O-glucuronide	0.017 ± 0.005	0.018 ± 0.005	0.007 ± 0.003	0.039 ± 0.010	0.17 ± 0.08
Naringenin 4-O-glucuronide	0.033 ± 0.012	0.035 ± 0.011	0.006 ± 0.003	0.083 ± 0.028	0.34 ± 0.18
Hesperetin 7-O-glucuronide	0.005 ± 0.002	0.007 ± 0.003	0.073 ± 0.033	0.68 ± 0.18	3.00 ± 0.53
Hesperetin 3-O-glucuronide	0.025 ± 0.007	0.064 ± 0.005	0.46 ± 0.16	5.10 ± 1.99	23.3 ± 3.7
Hesperetin 7-O-sulfate	0.002 ± 0.001	0.007 ± 0.000	0.033 ± 0.009	0.35 ± 0.10	1.52 ± 0.30
Hesperetin	0.0001 ± 0.0000	0.001 ± 0.000	0.001 ± 0.000	0.006 ± 0.002	0.092 ± 0.014
Catabolites, µmol					
2-Hydroxy-2-(4′ -hydroxyphenyl)acetic acid	1.38 ± 0.16	2.99 ± 0.34	2.77 ± 0.39	3.09 ± 0.35	13.7 ± 1.9
2-Hydroxy-2-(4′ -hydroxy-3′ -methoxyphenyl)acetic acid	1.09 ± 0.08	2.89 ± 0.23	1.76 ± 0.15	1.98 ± 0.27	9.38 ± 0.90
4′-Hydroxyhippuric acid	1.81 ± 0.24	3.65 ± 0.59	1.84 ± 0.19	3.51 ± 0.59	20.3 ± 2.5
3′-Hydroxyhippuric acid	5.14 ± 1.77	8.34 ± 3.40	1.89 ± 0.55	3.56 ± 2.28	31.9 ± 7.2
33-Hydroxy-3-(3′ -hydroxyphenyl)propanoic acid	19.6 ± 8.0	29.0 ± 13.9	7.36 ± 2.64	18.0 ± 12.8	114 ± 26
3-(3′-hydroxyphenyl)propanoic acid-4′-sulfate	0.23 ± 0.03	0.48 ± 0.07	0.15 ± 0.03	0.18 ± 0.05	1.52 ± 0.22
3-(3′-hydroxyphenyl)propanoic acid-4′-glucuronide	0.028 ± 0.003	0.066 ± 0.005	0.058 ± 0.005	0.053 ± 0.004	0.54 ± 0.23
3-(4′-hydroxyphenyl)propanoic acid-3′-sulfate	0.57 ± 0.23	0.35 ± 0.14	0.15 ± 0.06	0.22 ± 0.17	3.72 ± 1.58
Hippuric acid	24.4 ± 3.4	56.9 ± 9.2	40.0 ± 4.5	41.0 ± 6.4	193 ± 20
3-(4′-hydroxyphenyl)propanoic acid-3-glucuronide	0.14 ± 0.02	0.30 ± 0.04	0.15 ± 0.02	0.16 ± 0.03	1.28 ± 0.26
4′-Hydroxyphenylacetic acid	44.5 ± 5.3	117 ± 29	57.8 ± 10.5	63.5 ± 12.2	460 ± 71
3-(4-hydroxy-3′-methoxyphenyl)propanoic acid-4′-sulfate	0.50 ± 0.13	0.44 ± 0.09	0.10 ± 0.04	0.59 ± 0.42	3.91 ± 0.76
3-Phenylpropanoic acid-3′-sulfate	0.39 ± 0.15	0.60 ± 0.28	0.17 ± 0.06	0.32 ± 0.20	2.13 ± 0.45
3-Phenylpropanoic acid-4′-glucuronide	0.039 ± 0.013	0.060 ± 0.021	0.035 ± 0.006	0.046 ± 0.015	0.32 ± 0.11
3-(4-hydroxy 3′-methoxyphenyl)propanoic acid-4′-glucuronide	0.11 ± 0.03	0.11 ± 0.02	0.07 ± 0.01	0.12 ± 0.05	0.85 ± 0.12
3-Methoxy-4-hydroxybenzoic acid	0.43 ± 0.23	0.95 ± 0.47	0.30 ± 0.07	0.48 ± 0.10	5.83 ± 2.77
3-(3-hydroxy4′-methoxyphenyl)propanoic acid-3′-sulfate	0.016 ± 0.008	0.021 ± 0.012	0.006 ± 0.004	0.27 ± 0.13	3.75 ± 0.86
3′-Hydroxyphenylacetic acid	23.0 ± 4.5	58.8 ± 12.2	34.9 ± 6.3	32.2 ± 6.5	171 ± 32
4′ -Hydroxy-3′ -methoxyphenylacetic acid	5.19 ± 0.60	12.5 ± 1.6	6.86 ± 0.74	7.02 ± 1.03	40.5 ± 5.0
3-(3-hydroxy 4′-methoxyphenyl)propanoic-3′-glucuronide	0.002 ± 0.001	0.004 ± 0.002	0.003 ± 0.001	0.053 ± 0.022	0.62 ± 0.18
3-(4′-hydroxyphenyl)propanoic acid	0.005 ± 0.002	0.024 ± 0.009	0.014 ± 0.005	0.016 ± 0.004	0.089 ± 0.027
4′-Hydroxycinnamic acid	0.013 ± 0.008	0.017 ± 0.016	0.004 ± 0.002	0.057 ± 0.019	0.28 ± 0.16
3-(3′-hydroxyphenyl)propanoic acid	0.056 ± 0.011	0.11 ± 0.01	0.065 ± 0.010	0.17 ± 0.04	0.64 ± 0.09
3-(3′-methoxy-4′-hydroxyphenyl)propanoic acid	0.11 ± 0.05	0.16 ± 0.05	0.11 ± 0.03	0.32 ± 0.12	1.50 ± 0.49
3-(3′-hydroxy-4′-methoxyphenyl)propanoic acid	0.022 ± 0.003	0.049 ± 0.006	0.028 ± 0.003	0.049 ± 0.011	0.36 ± 0.06
3′,4′-dimethoxyphenylacetic acid	44.1 ± 13.6	48 ± 8	22.8 ± 2.0	30.3 ± 4.5	194 ± 21
4′ -Methoxycinnamic acid-3′ -glucuronide	0.054 ± 0.018	0.15 ± 0.08	0.06 ± 0.01	0.16 ± 0.06	1.78 ± 0.33
3-Hydroxy-3-(3′ -hydroxy-4′ -methoxyphenyl)propanoic acid	0.43 ± 0.14	0.48 ± 0.20	0.69 ± 0.26	7.93 ± 2.97	66.6 ± 12.8

Data expressed as mean ± standard deviation.

Table S3. Quantities of Hesperidin metabolites and Hesperidin catabolites in urine before and after Micronised Hesperidin epimeric mixture (MHEM) ingestion.

Variable	0 h	0-3 h	3-6 h	6-8 h	8-24 h
Metabolites, µmol					
Hesperetin 7,3'-di-O-glucuronide	0 ± 0	0.001 ± 0.001	0.041 ± 0.019	0.82 ± 0.23	4.66 ± 0.78
Naringenin 7-O-glucuronide	0.005 ± 0.002	0.009 ± 0.003	0.017 ± 0.006	0.11 ± 0.04	0.28 ± 0.06
Naringenin 4-O-glucuronide	0.007 ± 0.003	0.014 ± 0.006	0.016 ± 0.006	0.16 ± 0.03	0.49 ± 0.12
Hesperetin 7-O-glucuronide	0.004 ± 0.002	0.004 ± 0.002	0.075 ± 0.033	1.62 ± 0.47	4.24 ± 0.68
Hesperetin 3-O-glucuronide	0.019 ± 0.002	0.095 ± 0.013	0.49 ± 0.16	13.6 ± 4.2	37.7 ± 8.0
Hesperetin 7-O-sulfate	0.001 ± 0.000	0.010 ± 0.001	0.044 ± 0.015	0.95 ± 0.31	2.45 ± 0.53
Hesperetin	0 ± 0	0.001 ± 0.000	0.002 ± 0.000	0.014 ± 0.004	0.12 ± 0.02
Catabolites, µmol					
2-Hydroxy-2-(4′ -hydroxyphenyl)acetic acid	1.53 ± 0.15	3.28 ± 0.38	3.62 ± 0.48	3.02 ± 0.30	17.7 ± 3.2
2-Hydroxy-2-(4′ -hydroxy-3′ -methoxyphenyl)acetic acid	1.11 ± 0.09	2.49 ± 0.26	2.13 ± 0.30	2.41 ± 0.34	9.86 ± 0.60
4′-Hydroxyhippuric acid	2.05 ± 0.35	3.68 ± 0.52	3.14 ± 0.49	4.43 ± 0.52	18.1 ± 1.9
3′-Hydroxyhippuric acid	5.62 ± 2.74	9.62 ± 5.83	7.79 ± 5.82	6.04 ± 2.85	36.0 ± 10.6
33-Hydroxy-3-(3′ -hydroxyphenyl)propanoic acid	24.5 ± 14.9	39 ± 27	33.8 ± 26.6	24.6 ± 14.0	131 ± 47
3-(3′-hydroxyphenyl)propanoic acid-4′-sulfate	0.21 ± 0.02	0.34 ± 0.06	0.19 ± 0.04	0.25 ± 0.06	1.46 ± 0.18
3-(3′-hydroxyphenyl)propanoic acid-4′-glucuronide	0.024 ± 0.002	0.072 ± 0.007	0.073 ± 0.004	0.066 ± 0.012	0.25 ± 0.02
3-(4′-hydroxyphenyl)propanoic acid-3′-sulfate	0.27 ± 0.09	0.26 ± 0.08	0.14 ± 0.02	0.14 ± 0.04	1.21 ± 0.31
Hippuric acid	26.9 ± 4.2	66.4 ± 11.8	67.0 ± 10.4	48.6 ± 6.2	215 ± 16
3-(4′-hydroxyphenyl)propanoic acid-3-glucuronide	0.13 ± 0.02	0.30 ± 0.04	0.22 ± 0.03	0.21 ± 0.03	0.97 ± 0.06
4′-Hydroxyphenylacetic acid	51.5 ± 6.6	74.5 ± 17.9	64.3 ± 8.4	77.1 ± 11.8	439 ± 91
3-(4-hydroxy-3′-methoxyphenyl)propanoic acid-4′-sulfate	0.28 ± 0.07	0.46 ± 0.13	0.11 ± 0.05	0.37 ± 0.09	2.60 ± 0.52
3-Phenylpropanoic acid-3′-sulfate	0.45 ± 0.25	0.60 ± 0.34	0.60 ± 0.46	0.49 ± 0.25	2.57 ± 0.85
3-Phenylpropanoic acid-4′-glucuronide	0.065 ± 0.032	0.10 ± 0.04	0.087 ± 0.041	0.059 ± 0.022	0.40 ± 0.16
3-(4-hydroxy 3′-methoxyphenyl)propanoic acid-4′-glucuronide	0.060 ± 0.014	0.13 ± 0.03	0.10 ± 0.02	0.11 ± 0.02	0.62 ± 0.11
3-Methoxy-4-hydroxybenzoic acid	0.38 ± 0.15	0.42 ± 0.09	0.33 ± 0.06	0.67 ± 0.17	5.98 ± 3.96
3-(3-hydroxy4′-methoxyphenyl)propanoic acid-3′-sulfate	0.004 ± 0.002	0.0003 ± 0.0001	0.001 ± 0.000	0.47 ± 0.18	4.31 ± 0.87
3′-Hydroxyphenylacetic acid	22.7 ± 3.9	39.2 ± 7.1	40.1 ± 7.6	35.9 ± 7.3	159 ± 21
4′ -Hydroxy-3′ -methoxyphenylacetic acid	5.09 ± 0.48	9.66 ± 0.96	8.38 ± 0.96	7.98 ± 1.11	37.5 ± 2.6
3-(3-hydroxy 4′-methoxyphenyl)propanoic-3′-glucuronide	0.001 ± 0.000	0.001 ± 0.000	0.003 ± 0.001	0.09 ± 0.03	0.68 ± 0.15
3-(4′-hydroxyphenyl)propanoic acid	0.005 ± 0.001	0.016 ± 0.006	0.018 ± 0.009	0.008 ± 0.003	0.053 ± 0.025
4′-Hydroxycinnamic acid	0.010 ± 0.004	0.003 ± 0.002	0.004 ± 0.001	0.055 ± 0.024	0.084 ± 0.036
3-(3′-hydroxyphenyl)propanoic acid	0.15 ± 0.08	0.12 ± 0.04	0.12 ± 0.03	0.20 ± 0.03	0.76 ± 0.13
3-(3′-methoxy-4′-hydroxyphenyl)propanoic acid	0.076 ± 0.025	0.18 ± 0.05	0.15 ± 0.04	0.3 ± 0.1	0.92 ± 0.21
3-(3′-hydroxy-4′-methoxyphenyl)propanoic acid	0.026 ± 0.003	0.037 ± 0.004	0.035 ± 0.005	0.085 ± 0.035	0.37 ± 0.05
3′,4′-dimethoxyphenylacetic acid	23.3 ± 4.0	29.6 ± 3.0	29.4 ± 3.2	32.3 ± 3.7	183 ± 17
4′ -Methoxycinnamic acid-3′ -glucuronide	0.030 ± 0.006	0.052 ± 0.009	0.058 ± 0.007	0.29 ± 0.09	2.05 ± 0.30
3-Hydroxy-3-(3′ -hydroxy-4′ -methoxyphenyl)propanoic acid	0.18 ± 0.05	0.23 ± 0.05	0.53 ± 0.13	13.1 ± 3.8	87.5 ± 15.5

Data expressed as mean ± standard deviation.

Table S4. Quantities of Hesperidin metabolites and catabolites in urine before and after Micronised 2S-Hesperidin (M2SH) ingestion.

Variable	0 h	0-3 h	3-6 h	6-8 h	8-24 h
Metabolites, µmol					
Hesperetin 7,3'-di-O-glucuronide	0.004 ± 0.004	0.004 ± 0.003	0.069 ± 0.027	0.36 ± 0.09	1.77 ± 0.39
Naringenin 7-O-glucuronide	0.014 ± 0.003	0.025 ± 0.010	0.030 ± 0.009	0.077 ± 0.016	0.23 ± 0.06
Naringenin 4-O-glucuronide	0.024 ± 0.007	0.044 ± 0.019	0.063 ± 0.018	0.20 ± 0.04	0.38 ± 0.09
Hesperetin 7-O-glucuronide	0.006 ± 0.004	0.006 ± 0.003	0.14 ± 0.06	0.65 ± 0.15	1.55 ± 0.42
Hesperetin 3-O-glucuronide	0.062 ± 0.041	0.11 ± 0.02	0.74 ± 0.23	3.71 ± 1.02	15.0 ± 4.8
Hesperetin 7-O-sulfate	0.005 ± 0.004	0.010 ± 0.002	0.076 ± 0.028	0.36 ± 0.12	1.51 ± 0.53
Hesperetin	0.0002 ± 0.0001	0.001 ± 0.000	0.002 ± 0.000	0.009 ± 0.002	0.054 ± 0.013
Catabolites, µmol					
2-Hydroxy-2-(4′ -hydroxyphenyl)acetic acid	1.56 ± 0.20	3.72 ± 0.43	3.27 ± 0.45	3.45 ± 0.42	15.0 ± 1.4
2-Hydroxy-2-(4′ -hydroxy-3′ -methoxyphenyl)acetic acid	1.21 ± 0.12	2.64 ± 0.29	2.20 ± 0.32	2.50 ± 0.35	10.3 ± 1.0
4′-Hydroxyhippuric acid	2.49 ± 0.85	4.14 ± 0.63	3.15 ± 0.56	4.90 ± 0.71	17.6 ± 1.4
3′-Hydroxyhippuric acid	5.61 ± 3.25	10.2 ± 4.8	8.32 ± 5.42	10.6 ± 6.2	43.7 ± 8.9
33-Hydroxy-3-(3′ -hydroxyphenyl)propanoic acid	24.3 ± 16.4	34.8 ± 18.6	29.6 ± 19.6	40.0 ± 25.3	157 ± 40
3-(3′-hydroxyphenyl)propanoic acid-4′-sulfate	0.27 ± 0.04	0.43 ± 0.09	0.23 ± 0.05	0.29 ± 0.07	2.82 ± 1.37
3-(3′-hydroxyphenyl)propanoic acid-4′-glucuronide	0.036 ± 0.008	0.082 ± 0.009	0.072 ± 0.009	0.077 ± 0.012	1.95 ± 1.26
3-(4′-hydroxyphenyl)propanoic acid-3′-sulfate	0.35 ± 0.14	0.55 ± 0.26	0.22 ± 0.06	0.32 ± 0.15	3.50 ± 2.13
Hippuric acid	24.5 ± 4.0	65.2 ± 8.2	52 ± 6	61.7 ± 9.2	218 ± 11
3-(4′-hydroxyphenyl)propanoic acid-3-glucuronide	0.14 ± 0.01	0.31 ± 0.05	0.19 ± 0.02	0.26 ± 0.05	2.57 ± 1.05
4′-Hydroxyphenylacetic acid	44.1 ± 8.3	77.0 ± 17.4	53.0 ± 9.3	76.1 ± 17.6	385 ± 61
3-(4-hydroxy-3′-methoxyphenyl)propanoic acid-4′-sulfate	0.33 ± 0.08	0.63 ± 0.22	0.28 ± 0.10	0.36 ± 0.09	4.67 ± 1.53
3-Phenylpropanoic acid-3′-sulfate	0.56 ± 0.35	0.79 ± 0.32	0.56 ± 0.34	1.07 ± 0.70	3.33 ± 0.74
3-Phenylpropanoic acid-4′-glucuronide	0.054 ± 0.038	0.070 ± 0.024	0.080 ± 0.036	0.12 ± 0.07	0.48 ± 0.20
3-(4-hydroxy 3′-methoxyphenyl)propanoic acid-4′-glucuronide	0.066 ± 0.011	0.14 ± 0.03	0.11 ± 0.02	0.11 ± 0.02	1.19 ± 0.47
3-Methoxy-4-hydroxybenzoic acid	0.23 ± 0.12	0.58 ± 0.17	0.36 ± 0.08	0.68 ± 0.22	13.5 ± 6.1
3-(3-hydroxy4′-methoxyphenyl)propanoic acid-3′-sulfate	0.14 ± 0.14	0.05 ± 0.04	0.13 ± 0.05	1.17 ± 0.44	10.7 ± 2.1
3′-Hydroxyphenylacetic acid	24.1 ± 3.4	46.9 ± 7.4	39.8 ± 5.3	36.4 ± 5.3	182 ± 29
4′ -Hydroxy-3′ -methoxyphenylacetic acid	6.22 ± 0.68	12.1 ± 1.3	9.16 ± 0.93	9.10 ± 0.94	56.1 ± 16.6
3-(3-hydroxy 4′-methoxyphenyl)propanoic-3′-glucuronide	0.012 ± 0.010	0.006 ± 0.003	0.024 ± 0.009	0.18 ± 0.06	1.51 ± 0.32
3-(4′-hydroxyphenyl)propanoic acid	0.022 ± 0.016	0.025 ± 0.011	0.029 ± 0.011	0.016 ± 0.005	0.069 ± 0.025
4′-Hydroxycinnamic acid	0.018 ± 0.011	0.012 ± 0.006	0.006 ± 0.003	0.043 ± 0.011	0.10 ± 0.03
3-(3′-hydroxyphenyl)propanoic acid	0.065 ± 0.016	0.12 ± 0.02	0.095 ± 0.016	0.20 ± 0.02	0.76 ± 0.15
3-(3′-methoxy-4′-hydroxyphenyl)propanoic acid	0.078 ± 0.046	0.21 ± 0.06	0.31 ± 0.15	0.27 ± 0.07	1.09 ± 0.22
3-(3′-hydroxy-4′-methoxyphenyl)propanoic acid	0.025 ± 0.004	0.041 ± 0.006	0.031 ± 0.006	0.091 ± 0.034	0.55 ± 0.08
3′,4′-dimethoxyphenylacetic acid	23.5 ± 4.6	32.9 ± 3.1	25.0 ± 3.2	32.9 ± 2.9	182 ± 18
4′ -Methoxycinnamic acid-3′ -glucuronide	0.093 ± 0.063	0.12 ± 0.04	0.13 ± 0.03	0.50 ± 0.17	4.01 ± 0.73
3-Hydroxy-3-(3′ -hydroxy-4′ -methoxyphenyl)propanoic acid	1.57 ± 1.31	1.16 ± 0.59	3.63 ± 1.73	23.8 ± 7.7	155 ± 23

Data expressed as mean ± standard deviation.

Table S5. Concentration of Hesperidin metabolites and catabolites in serum before and after Hesperidin epimeric mixture (HEM) ingestion.

Variable	0 h	2 h	3 h	4 h	5 h	6 h	7 h	8 h	24 h
Metabolites, µM									
Hesperetin, 7,3,di-O-Glucuronide	0.001 ± 0.000	0.001 ± 0.000	0.001 ± 0.000	0.002 ± 0.004	0.004 ± 0.009	0.014 ± 0.017	0.029 ± 0.025	0.040 ± 0.029	0.013 ± 0.021
Hesperetin 7-O-Glucuronide	0.0003 ± 0.0000	0.0003 ± 0.0000	0.0003 ± 0.0000	0.003 ± 0.007	0.018 ± 0.024	0.054 ± 0.059	0.083 ± 0.070	0.084 ± 0.052	0.028 ± 0.055
Hesperetin 3-O-Glucuronide	0.001 ± 0.000	0.001 ± 0.000	0.003 ± 0.008	0.012 ± 0.017	0.030 ± 0.039	0.085 ± 0.096	0.13 ± 0.12	0.15 ± 0.12	0.057 ± 0.086
Hesperetin 7-O-Sulfate	0.0001 ± 0.0003	0.0003 ± 0.0005	0.001 ± 0.002	0.004 ± 0.007	0.012 ± 0.016	0.085 ± 0.084	0.086 ± 0.065	0.086 ± 0.049	0.026 ± 0.042
Hesperetin	0.0003 ± 0.0008	0.0001 ± 0.0000	0.0001 ± 0.0000	0.0001 ± 0.0000	0.0001 ± 0.0000	0.001 ± 0.001	0.0004 ± 0.0010	0.001 ± 0.001	0.0001 ± 0.0001
Catabolites, µM									
2-Hydroxy-2-(4′ -hydroxy-3′ -methoxyphenyl)acetic acid	0.028 ± 0.007	0.024 ± 0.008	0.024 ± 0.007	0.024 ± 0.008	0.025 ± 0.007	0.028 ± 0.008	0.025 ± 0.005	0.024 ± 0.008	0.029 ± 0.008
4'-Hydroxyhippuric acid	0.022 ± 0.008	0.016 ± 0.008	0.014 ± 0.007	0.014 ± 0.007	0.014 ± 0.007	0.028 ± 0.009	0.026 ± 0.007	0.022 ± 0.006	0.034 ± 0.023
3'-Hydroxyhippuric acid	0.065 ± 0.090	0.040 ± 0.058	0.030 ± 0.037	0.024 ± 0.028	0.017 ± 0.017	0.019 ± 0.014	0.028 ± 0.017	0.032 ± 0.021	0.11 ± 0.07
3-Hydroxy-3-(3′ -hydroxyphenyl)propanoic acid	0.11 ± 0.24	0.035 ± 0.088	0.029 ± 0.065	0.021 ± 0.058	0.017 ± 0.042	0.026 ± 0.053	0.028 ± 0.048	0.045 ± 0.063	0.27 ± 0.24
Hippuric acid	1.18 ± 0.82	0.69 ± 0.49	0.59 ± 0.42	0.57 ± 0.39	0.56 ± 0.37	0.63 ± 0.29	0.58 ± 0.28	0.53 ± 0.25	1.44 ± 0.69
4'-Hydroxyphenylacetic acid	4.40 ± 5.29	3.83 ± 3.59	3.35 ± 2.75	3.04 ± 2.59	2.64 ± 1.52	3.54 ± 2.08	3.51 ± 2.11	3.30 ± 1.97	4.78 ± 4.03
3-(4'-hydroxy-3'-methoxyphenyl)propanoic acid -4'-sulfate	0.006 ± 0.005	0.003 ± 0.002	0.002 ± 0.002	0.002 ± 0.001	0.002 ± 0.002	0.004 ± 0.004	0.005 ± 0.004	0.005 ± 0.003	0.019 ± 0.035
3-(4'-hydroxy-3'-methoxyphenyl)propanoic acid -4'-glucuronide	0.003 ± 0.002	0.002 ± 0.001	0.002 ± 0.001	0.001 ± 0.001	0.001 ± 0.001	0.002 ± 0.002	0.003 ± 0.002	0.003 ± 0.002	0.008 ± 0.009
3-(4'-hydroxy-3'-methoxyphenyl)propanoic acid -3'-sulfate	0.001 ± 0.001	0.0004 ± 0.0012	0.0004 ± 0.0012	0.001 ± 0.001	0.002 ± 0.003	0.006 ± 0.008	0.016 ± 0.018	0.022 ± 0.020	0.024 ± 0.026
3'-hydroxyphenylacetic acid	3.67 ± 1.91	3.37 ± 1.74	3.19 ± 1.75	2.92 ± 1.57	2.57 ± 1.43	3.04 ± 1.50	2.95 ± 1.38	2.91 ± 1.07	3.1 ± 1.3
4′ -Hydroxy-3′ -methoxyphenylacetic acid	0.23 ± 0.09	0.17 ± 0.09	0.15 ± 0.08	0.15 ± 0.07	0.15 ± 0.08	0.16 ± 0.05	0.15 ± 0.08	0.13 ± 0.07	0.22 ± 0.07
3-(3'-hydroxy-4'-methoxyphenyl)propanoic acid -3'-glucuronide	0.0002 ± 0.0002	0.0001 ± 0.0002	0.0001 ± 0.0001	0.0001 ± 0.0001	0.0002 ± 0.0002	0.001 ± 0.001	0.001 ± 0.002	0.002 ± 0.002	0.002 ± 0.002
3-(3'-hydroxyphenyl)propanoic acid	0.42 ± 0.10	0.42 ± 0.09	0.40 ± 0.08	0.37 ± 0.08	0.36 ± 0.09	0.45 ± 0.08	0.48 ± 0.09	0.46 ± 0.09	0.41 ± 0.14
3-(4'-hydroxy-3'-methoxyphenyl)propanoic acid	0.026 ± 0.019	0.015 ± 0.010	0.010 ± 0.006	0.009 ± 0.007	0.011 ± 0.010	0.028 ± 0.027	0.034 ± 0.028	0.03 ± 0.02	0.086 ± 0.141
3-(3'-hydroxyphenyl-4'-methoxyphenyl) propionic acid	0.010 ± 0.021	0.007 ± 0.015	0.007 ± 0.016	0.016 ± 0.024	0.041 ± 0.059	0.18 ± 0.23	0.31 ± 0.33	0.35 ± 0.31	0.2 ± 0.2
3 Phenylpropanoic acid	0.49 ± 0.29	0.50 ± 0.37	0.45 ± 0.31	0.38 ± 0.27	0.34 ± 0.24	0.50 ± 0.38	0.50 ± 0.39	0.47 ± 0.35	0.73 ± 0.47
4′ -Methoxycinnamic acid-3′ -glucuronide	0.001 ± 0.001	0.001 ± 0.001	0.001 ± 0.001	0.0004 ± 0.0005	0.0004 ± 0.0005	0.001 ± 0.001	0.002 ± 0.002	0.003 ± 0.003	0.007 ± 0.008

Data expressed as mean ± standard deviation.

Table S6. Concentration of Hesperidin metabolites and catabolites in serum before and after Micronised Hesperidin epimeric mixture (MHEM) ingestion.

Variable	0 h	2 h	3 h	4 h	5 h	6 h	7 h	8 h	24 h
Metabolites, µM									
Hesperetin, 7,3,di-O-Glucuronide	0.001 ± 0.000	0.001 ± 0.000	0.001 ± 0.000	0.001 ± 0.002	0.001 ± 0.004	0.018 ± 0.021	0.061 ± 0.060	0.061 ± 0.056	0.026 ± 0.038
Hesperetin 7-O-Glucuronide	0.0005 ± 0.0009	0.001 ± 0.002	0.001 ± 0.003	0.003 ± 0.005	0.021 ± 0.027	0.092 ± 0.091	0.17 ± 0.16	0.14 ± 0.13	0.046 ± 0.075
Hesperetin 3-O-Glucuronide	0.001 ± 0.000	0.001 ± 0.000	0.001 ± 0.000	0.008 ± 0.016	0.037 ± 0.041	0.11 ± 0.08	0.27 ± 0.27	0.22 ± 0.20	0.071 ± 0.094
Hesperetin 7-O-Sulfate	0.0001 ± 0.0002	0.001 ± 0.000	0.001 ± 0.001	0.005 ± 0.008	0.022 ± 0.036	0.16 ± 0.20	0.28 ± 0.31	0.21 ± 0.20	0.054 ± 0.078
Hesperetin	0.0001 ± 0.0000	0.0001 ± 0.0000	0.0001 ± 0.0000	0.0001 ± 0.0000	0.0002 ± 0.0006	0.002 ± 0.003	0.003 ± 0.005	0.002 ± 0.003	0.001 ± 0.001
Catabolites, µM									
2-Hydroxy-2-(4′ -hydroxy-3′ -methoxyphenyl)acetic acid	0.029 ± 0.007	0.027 ± 0.007	0.024 ± 0.007	0.023 ± 0.009	0.025 ± 0.007	0.028 ± 0.008	0.023 ± 0.010	0.024 ± 0.005	0.029 ± 0.006
4'-Hydroxyhippuric acid	0.026 ± 0.010	0.019 ± 0.008	0.016 ± 0.006	0.014 ± 0.005	0.015 ± 0.005	0.034 ± 0.014	0.032 ± 0.015	0.025 ± 0.005	0.033 ± 0.011
3'-Hydroxyhippuric acid	0.048 ± 0.046	0.034 ± 0.031	0.031 ± 0.025	0.024 ± 0.018	0.021 ± 0.017	0.029 ± 0.020	0.044 ± 0.032	0.062 ± 0.054	0.14 ± 0.24
3-Hydroxy-3-(3′ -hydroxyphenyl)propanoic acid	0.073 ± 0.141	0.028 ± 0.060	0.033 ± 0.058	0.039 ± 0.052	0.032 ± 0.055	0.025 ± 0.051	0.070 ± 0.119	0.11 ± 0.18	0.38 ± 0.77
Hippuric acid	1.15 ± 0.49	0.76 ± 0.37	0.66 ± 0.28	0.61 ± 0.22	0.58 ± 0.19	0.71 ± 0.21	0.64 ± 0.24	0.67 ± 0.27	1.58 ± 1.60
4'-Hydroxyphenylacetic acid	4.06 ± 3.71	3.69 ± 3.04	3.31 ± 2.43	3.06 ± 2.09	2.71 ± 1.85	3.99 ± 2.36	4.08 ± 3.30	4.18 ± 3.94	4.17 ± 3.34
3-(4'-hydroxy-3'-methoxyphenyl)propanoic acid -4'-sulfate	0.007 ± 0.006	0.005 ± 0.005	0.003 ± 0.003	0.002 ± 0.003	0.002 ± 0.003	0.005 ± 0.005	0.006 ± 0.006	0.006 ± 0.003	0.010 ± 0.008
3-(4'-hydroxy-3'-methoxyphenyl)propanoic acid -4'-glucuronide	0.003 ± 0.004	0.003 ± 0.004	0.002 ± 0.003	0.002 ± 0.002	0.002 ± 0.002	0.003 ± 0.003	0.004 ± 0.004	0.004 ± 0.003	0.006 ± 0.006
3-(4'-hydroxy-3'-methoxyphenyl)propanoic acid -3'-sulfate	0.0001 ± 0.0003	0.0002 ± 0.0005	0.0002 ± 0.0004	0.0002 ± 0.0005	0.001 ± 0.002	0.007 ± 0.008	0.029 ± 0.029	0.041 ± 0.041	0.022 ± 0.036
3'-hydroxyphenylacetic acid	3.44 ± 1.53	3.38 ± 1.36	3.24 ± 1.63	2.94 ± 1.39	2.54 ± 1.01	3.20 ± 1.37	3.47 ± 1.34	3.22 ± 1.15	3.37 ± 1.75
4′ -Hydroxy-3′ -methoxyphenylacetic acid	0.24 ± 0.10	0.19 ± 0.10	0.17 ± 0.08	0.16 ± 0.06	0.15 ± 0.07	0.19 ± 0.09	0.19 ± 0.10	0.17 ± 0.14	0.23 ± 0.11
3-(3'-hydroxy-4'-methoxyphenyl)propanoic acid -3'-glucuronide	0.0001 ± 0.0001	0.0001 ± 0.0001	0.0001 ± 0.0001	0.0001 ± 0.0001	0.0001 ± 0.0002	0.001 ± 0.001	0.002 ± 0.003	0.004 ± 0.005	0.002 ± 0.002
3-(3'-hydroxyphenyl)propanoic acid	0.44 ± 0.17	0.46 ± 0.21	0.43 ± 0.19	0.40 ± 0.21	0.35 ± 0.15	0.50 ± 0.17	0.52 ± 0.15	0.49 ± 0.22	0.49 ± 0.17
3-(4'-hydroxy-3'-methoxyphenyl)propanoic acid	0.033 ± 0.036	0.026 ± 0.030	0.018 ± 0.020	0.014 ± 0.015	0.015 ± 0.018	0.034 ± 0.032	0.044 ± 0.036	0.035 ± 0.020	0.063 ± 0.069
3-(3'-hydroxyphenyl-4'-methoxyphenyl) propionic acid	0.004 ± 0.005	0.004 ± 0.006	0.004 ± 0.006	0.010 ± 0.016	0.038 ± 0.060	0.22 ± 0.24	0.70 ± 0.65	0.74 ± 0.74	0.29 ± 0.52
3 Phenylpropanoic acid	0.50 ± 0.19	0.50 ± 0.24	0.43 ± 0.19	0.35 ± 0.14	0.30 ± 0.12	0.49 ± 0.21	0.53 ± 0.28	0.53 ± 0.28	0.72 ± 0.46
4′ -Methoxycinnamic acid-3′ -glucuronide	0.0006 ± 0.0007	0.0005 ± 0.0005	0.0003 ± 0.0003	0.0002 ± 0.0002	0.0003 ± 0.0003	0.001 ± 0.001	0.004 ± 0.004	0.005 ± 0.005	0.006 ± 0.006

Data expressed as mean ± standard deviation.

Table S7. Concentration of Hesperidin metabolites and catabolites in serum before and after Micronised 2S-Hesperidin (M2SH) ingestion.

Variable	0 h	2 h	3 h	4 h	5 h	6 h	7 h	8 h	24 h
Metabolites, µM									
Hesperetin, 7,3,di-O-Glucuronide	0.001 ± 0.000	0.001 ± 0.000	0.001 ± 0.000	0.003 ± 0.006	0.009 ± 0.012	0.016 ± 0.019	0.030 ± 0.029	0.035 ± 0.024	0.007 ± 0.015
Hesperetin 7-O-Glucuronide	0.0004 ± 0.0005	0.0005 ± 0.0009	0.002 ± 0.004	0.008 ± 0.014	0.022 ± 0.033	0.032 ± 0.034	0.049 ± 0.045	0.054 ± 0.043	0.009 ± 0.020
Hesperetin 3-O-Glucuronide	0.005 ± 0.010	0.003 ± 0.008	0.009 ± 0.017	0.015 ± 0.032	0.041 ± 0.056	0.074 ± 0.075	0.15 ± 0.14	0.18 ± 0.17	0.029 ± 0.061
Hesperetin 7-O-Sulfate	0.001 ± 0.003	0.001 ± 0.001	0.003 ± 0.006	0.011 ± 0.019	0.027 ± 0.043	0.055 ± 0.071	0.1 ± 0.1	0.12 ± 0.12	0.023 ± 0.050
Hesperetin	0.0001 ± 0.0000	0.0001 ± 0.0000	0.0001 ± 0.0000	0.0003 ± 0.0009	0.0003 ± 0.0008	0.0006 ± 0.0014	0.001 ± 0.002	0.001 ± 0.002	0.0002 ± 0.0007
Catabolites, µM									
2-Hydroxy-2-(4′ -hydroxy-3′ -methoxyphenyl)acetic acid	0.027 ± 0.009	0.022 ± 0.007	0.024 ± 0.005	0.025 ± 0.007	0.023 ± 0.009	0.029 ± 0.006	0.028 ± 0.011	0.025 ± 0.007	0.032 ± 0.009
4'-Hydroxyhippuric acid	0.024 ± 0.009	0.017 ± 0.008	0.017 ± 0.008	0.017 ± 0.009	0.015 ± 0.007	0.032 ± 0.010	0.034 ± 0.013	0.028 ± 0.012	0.037 ± 0.025
3'-Hydroxyhippuric acid	0.057 ± 0.066	0.046 ± 0.044	0.043 ± 0.043	0.037 ± 0.035	0.035 ± 0.037	0.045 ± 0.056	0.062 ± 0.080	0.069 ± 0.065	0.13 ± 0.17
3-Hydroxy-3-(3′ -hydroxyphenyl)propanoic acid	0.11 ± 0.15	0.046 ± 0.083	0.066 ± 0.167	0.069 ± 0.171	0.083 ± 0.179	0.13 ± 0.22	0.12 ± 0.19	0.15 ± 0.18	0.36 ± 0.57
Hippuric acid	1.23 ± 0.78	0.76 ± 0.49	0.73 ± 0.48	0.74 ± 0.42	0.75 ± 0.38	0.85 ± 0.35	0.85 ± 0.51	0.82 ± 0.61	1.39 ± 0.79
4'-Hydroxyphenylacetic acid	2.66 ± 1.13	2.57 ± 0.99	2.68 ± 1.36	2.49 ± 1.44	2.55 ± 1.24	3.69 ± 1.35	3.90 ± 1.69	3.56 ± 1.37	3.34 ± 1.43
3-(4'-hydroxy-3'-methoxyphenyl)propanoic acid -4'-sulfate	0.078 ± 0.258	0.016 ± 0.039	0.005 ± 0.005	0.003 ± 0.004	0.003 ± 0.003	0.004 ± 0.003	0.007 ± 0.006	0.008 ± 0.007	0.035 ± 0.102
3-(4'-hydroxy-3'-methoxyphenyl)propanoic acid -4'-glucuronide	0.005 ± 0.007	0.003 ± 0.002	0.003 ± 0.002	0.002 ± 0.002	0.002 ± 0.001	0.002 ± 0.002	0.004 ± 0.002	0.004 ± 0.003	0.017 ± 0.050
3-(4'-hydroxy-3'-methoxyphenyl)propanoic acid -3'-sulfate	0.002 ± 0.005	0.002 ±0.006	0.002 ± 0.005	0.004 ± 0.009	0.014 ± 0.025	0.028 ± 0.045	0.058 ± 0.066	0.10 ± 0.07	0.028 ± 0.046
3'-hydroxyphenylacetic acid	3.36 ± 1.51	3.25 ± 1.30	3.27 ± 1.58	2.90 ± 1.46	2.61 ± 1.30	3.15 ± 1.35	3.51 ± 1.55	3.46 ± 1.45	2.95 ± 1.49
4′ -Hydroxy-3′ -methoxyphenylacetic acid	0.25 ± 0.12	0.18 ± 0.11	0.16 ± 0.11	0.16 ± 0.09	0.18 ± 0.10	0.20 ± 0.09	0.19 ± 0.14	0.18 ± 0.13	0.28 ± 0.28
3-(3'-hydroxy-4'-methoxyphenyl)propanoic acid -3'-glucuronide	0.0002 ± 0.0003	0.0002 ± 0.0004	0.0002 ± 0.0003	0.0003 ± 0.0005	0.001 ± 0.001	0.002 ± 0.003	0.004 ± 0.004	0.007 ± 0.005	0.003 ± 0.003
3-(3'-hydroxyphenyl)propanoic acid	0.46 ± 0.22	0.47 ± 0.23	0.45 ± 0.18	0.42 ± 0.18	0.38 ± 0.16	0.54 ± 0.18	0.58 ± 0.19	0.54 ± 0.19	0.50 ± 0.22
3-(4'-hydroxy-3'-methoxyphenyl)propanoic acid	0.034 ± 0.029	0.029 ± 0.031	0.021 ± 0.021	0.017 ± 0.017	0.017 ± 0.014	0.028 ± 0.017	0.056 ± 0.050	0.053 ± 0.051	0.17 ± 0.48
3-(3'-hydroxyphenyl-4'-methoxyphenyl) propionic acid	0.009 ± 0.013	0.009 ± 0.019	0.027 ± 0.073	0.10 ± 0.28	0.26 ± 0.50	0.57 ± 0.94	1.09 ± 1.21	1.41 ± 1.14	0.39 ± 0.73
3 Phenylpropanoic acid	0.51 ± 0.32	0.58 ± 0.37	0.51 ± 0.36	0.47 ± 0.30	0.41 ± 0.27	0.56 ± 0.32	0.63 ± 0.41	0.60 ± 0.38	0.71 ± 0.41
4′ -Methoxycinnamic acid-3′ -glucuronide	0.001 ± 0.002	0.001 ± 0.002	0.001 ± 0.002	0.001 ± 0.001	0.002 ± 0.002	0.003 ± 0.003	0.005 ± 0.004	0.007 ± 0.005	0.008 ± 0.010

Data expressed as mean ± standard deviation

Table S8. Concentration of Hesperidin metabolites plus Hesperidin catabolites (nM) in serum before and after Hesperidin epimeric mixture (HEM), Micronised Hesperidin epimeric mixture (MHEM) and Micronised 2S-Hesperidin (M2SH) treatments.

	Time after ingestion									
**Intervention**	**Baseline (0 h)**	**2 h**	**3 h**	**4 h**	**5 h**	**6 h**	**7 h**	**8 h**	**24 h**	**P for trend**
HEM	126	**45**[*]	41	58	119	**448**[*]	**676**[†]	**766**[‡]	**624**[‡]	**<0.001**[a]
(n=13)	(65)	**(24)**	(18)	(19)	(33)	**(120)**	**(142)**	**(113)**	**(60)**	[b] **0.008**
MHEM	79	34	40	66	**254**[†]	**650**[‡]	**1612**[‡]	**1545**[‡]	**913**[†]	**<0.001**[a]
(n=14)	(37)	(16)	(15)	(16)	**(42)**	**(162)**	**(354)**	**(339)**	**(283)**	0.829
2SH	123	63	111	215	**465**[*]	**917**[†]	**1619**[‡]	**2084**[‡]	**854**[†]	**<0.001**[a]
(n=13)	(43)	(29)	(72)	(139)	**(217)**	**(336)**	**(398)**	**(327)**	**(298)**	0.840

Data expressed as mean (standard error). Metabolites involved: Hesperetin (Hesp), 7, 3' di-O- glucuronide (gluc)+ Hesp 7-O-gluc + Hesp 3-O-gluc + Hesp 7-O-sulfate+ Hesp. Catabolites involved: isoferulic acid-3'-glucuronide + 3-(3'- hydroxy-4'-methoxyphenyl) propionic acid (Dihydro-isoferulic acid) + 3-(3'- hydroxy 4'-methoxyphenyl) propionic acid-3'-sulfate + 3-(3'- hydroxy 4'-methoxyphenyl) propionic acid-3'-O-glucuronide + 3'-(3'-hydroxyphenyl) hydracrylic acid. General Linear Model with Bonferroni correction. Data log transformed for normalization.[*] P<0.05,[†] P<0.01, [‡] P<0.001 *versus* baseline. P for trend: a linear, [b] quadratic. Significant results in **bold.**

Table S9. Concentration of Hesperidin metabolites (nM) in serum before and after Hesperidin epimeric mixture (HEM), Micronised Hesperidin epimeric mixture (MHEM) and Micronised 2S-Hesperidin (M2SH) treatments.

	Time after ingestion									
**Intervention**	**Baseline (0 h)**	**2 h**	**3 h**	**4 h**	**5 h**	**6 h**	**7 h**	**8 h**	**24 h**	**P for trend**
HEM (n=14)	1.64	1.58	4.29	20.9	***63.4***	**239**[‡]	**332**[‡]	**361**[‡]	**124**[‡]	**[a]<0.001**
	(0.25)	(0.13)	(2.57)	(8.10)	***(21.4)***	**(63.3)**	**(65.1)**	**(58.1)**	**(54)**	[b] 0.173
MHEM (n=14)	1.64	**2.29**[*]	3.09	16.6	**81.9**[†]	**381**[‡]	**785**[‡]	**635**[‡]	**198**[†]	**[a]<0.001**
	(0.24)	**(0.50)**	(0.82)	(7.8)	**(26.6)**	**(102)**	**(201)**	**(144)**	**(73.6)**	**[b] 0.038**
2SH (n=14)	6.50	4.42	1.46	35.9	99.7	**177**[‡]	**329**[‡]	**393**[‡]	67.8	**[a]<0.001**
	(3.50)	(2.4)	(7.2)	(18.5)	(37.1)	**(50.8)**	**(81.7)**	**(89.8)**	(38.6)	**[b]0.002**

Data expressed as mean (standard error). Metabolites involved: Hesperetin (Hesp), 7, 3' di-O- glucuronide (gluc)+ Hesp 7-O-gluc + Hesp 3-O-gluc + Hesp 7-O-sulfate+ Hesp. General Linear Model with Bonferroni correction. Data log transformed for normalization. [*] P<0.05, [†] P<0.01, [‡] P<0.001*versus* baseline. P for trend: [a] linear, [b] quadratic.

Table S10. Concentration of Hesperidin catabolites (nM) in serum before and after Hesperidin epimeric mixture (HEM), Micronised Hesperidin epimeric mixture (MHEM) and Micronised 2S-Hesperidin (M2SH) treatments.

	Time after ingestion									
**Intervention**	**Baseline (0 h)**	**2 h**	**3 h**	**4 h**	**5 h**	**6 h**	**7 h**	**8 h**	**24 h**	**P for trend**
HEM (n=13)	124	43	37	38	60	215	357	421	**495***	**<0.001[a]**
	(65)	(25)	(18)	(16)	(19)	(67*)*	(97)	(89)	**(80)**	[b] 0.119
MHEM (n=14)	77	32	37	49	71	253	**801***	**903***	**698***	**<0.001[a]**
	(37)	(16)	(15)	(13)	(22)	(75)	**(190)**	**(210)**	**(235)**	0.124
2SH (n=13)	119	59	96	177	358	718	**1281***	**1672***	782	**<0.001[a]**
	(43)	(27)	(67)	(126)	(193)	(317)	**(390)**	**(348**)	(282)	0.413

Data expressed as mean (standard error). Catabolites involved: isoferulic acid-3'-glucuronide + 3-(3'- hydroxy- 4'-methoxyphenyl) propionic acid (Dihydro-isoferulic acid) + 3-(3'- hydroxy 4'-methoxyphenyl) propionic acid-3'-sulfate + 3-(3'-hydroxy 4'-methoxyphenyl) propionic acid-3'-O-glucuronide + 3'-(3'-hydroxyphenyl) hydracrylic acid . General Linear Model with Bonferroni correction. Data log transformed for normalization.* P<0.05 *versus* baseline. P for trend: a linear, [b] quadratic. Significant results in **bold**.
